# Lassa virus activates myeloid dendritic cells but suppresses their ability to stimulate T cells

**DOI:** 10.1371/journal.ppat.1007430

**Published:** 2018-11-12

**Authors:** Justine Schaeffer, Xavier Carnec, Stéphanie Reynard, Mathieu Mateo, Caroline Picard, Natalia Pietrosemoli, Marie-Agnès Dillies, Sylvain Baize

**Affiliations:** 1 Unité de Biologie des Infections Virales Emergentes, Institut Pasteur; Centre International de Recherche en Infectiologie (INSERM, CNRS, ENS Lyon, Université Lyon I), Lyon, France; 2 Bioinformatics and Biostatistics Hub, Centre de Bioinformatique Biostatistique et Biologie Intégrative (C3BI, USR 3756, IP CNRS), Institut Pasteur, Paris, France; University Hospital Center and University of Lausanne, SWITZERLAND

## Abstract

Lassa virus (LASV) is responsible for a viral hemorrhagic fever in humans and the death of 3,000 to 5,000 people every year. The immune response to LASV is poorly understood, but type I interferon (IFN-I) and T-cell responses appear to be critical for the host. We studied the response of myeloid dendritic cells (mDC) to LASV, as mDCs are involved in both IFN-I production and T-cell activation. We compared the response of primary human mDCs to LASV and Mopeia virus (MOPV), which is similar to LASV, but non-pathogenic. We showed that mDCs produced substantial amounts of IFN-I in response to both LASV and MOPV. However, only MOPV-infected mDCs were able to activate T cells. More surprisingly, coculture with T cells completely inhibited the activation of LASV-infected mDCs. These differences between LASV and MOPV were mostly due to the LASV nucleoprotein, which has major immunosuppressive properties, but the glycoprotein was also involved. Overall, these results suggest that mDCs may be important for the global response to LASV and play a role in the outcome of Lassa fever.

## Author summary

Lassa fever is a viral hemorrhagic fever and a major public health issue in West Africa. Lassa virus, the causative agent of Lassa fever, is listed by the World Health Organization as one of the emerging pathogens likely to cause severe outbreaks in the near future. Indeed, there is currently no vaccine and no treatment against Lassa virus. Determinants of Lassa virus high pathogenicity are not completely understood. However, it has been shown that rapid type I interferon response and efficient T cell response were critical to survive Lassa fever. Dendritic cells are at the crossroads of innate and adaptive immunity. Their direct response to viral infection includes type I interferon production. They can also present viral antigens, initiating the T cell responses. We decided to investigate how dendritic cells respond to Lassa virus to evaluate their importance in the global immune response. We showed that primary human myeloid dendritic cells are activated by Lassa virus infection, and produce type I interferon. However, Lassa virus-infected dendritic cells were not able to activate T cells. We also elucidated the roles of viral proteins in the modulation of dendritic cell responses.

## Introduction

Viral hemorrhagic fevers (VHF) are an important global health problem with a significant yearly death toll. Among VHFs, Lassa fever (LF) is endemic in West Africa, with an estimated 300,000 to 500,000 cases and 3,000 to 5,000 deaths every year [1]. Populations living from Senegal to Nigeria are at risk, which is up to 200 million people [2]. Lassa virus (LASV), the causative agent of LF, is listed by the World Health Organization (WHO) as one of the emerging pathogens likely to cause severe outbreaks in the near future, and for which there are few or no medical countermeasures [3]. There are currently no approved vaccines against LASV, no treatment efficient in endemic areas, and limited knowledge about the pathogenesis of LF.

LASV is an Old-World Arenavirus. This enveloped virus contains two segments of negative strand RNA (S and L) coding for four proteins. The nucleoprotein (NP) and glycoprotein precursor (GPC) are coded by the S segment, the matrix protein (Z) and RNA-dependent RNA polymerase (L) by the L segment. ORFs are coded in ambisense and separated by a hairpin structure containing termination sites.

LF begins as a non-specific febrile illness, indiscernible from multiple diseases more common in West Africa, such as malaria, typhoid, dysentery, arboviruses, etc [4]. LF then progresses to pharyngitis, vomiting, diarrhea, and hemorrhagic symptoms. Hypovolemia, multiple organ failure, and shock syndrome then lead to death [5]. The pathophysiological mechanisms that give rise to these symptoms are still poorly understood, but adverse effects of the immune response are strongly suspected [6]. In humans, only small amounts of neutralizing antibodies are detected late after the disease and the humoral response does not correlate with survival [7]. Recovery from LF appears to be associated with early innate immunity and effective T-cell responses [8]. Studies with non-human primates (NHPs) support this hypothesis, in which the survival of LASV-infected NHPs correlates with the early type I interferon (IFN-I) response and detection of circulating antigen-experienced T cells [9].

Early innate responses and the initiation of T-cell immunity are two properties of dendritic cells (DC). DCs are also important targets of LASV *in vitro* and *in vivo* [10,11]. Among DCs, plasmacytoid dendritic cells (pDC) are highly potent IFN-I producers [12], whereas myeloid dendritic cells (mDC) are specialized in antigen presentation and the induction of T-cell responses [13]. However, mDCs can also produce IFN-I under certain conditions, which can play a crucial role in i*n vivo*, as they are more numerous that pDCs [14]. In mice infected with Lymphocytic Choriomeningitis Virus (LCMV), mDCs are the main source of IFN-I, and such IFN-I production is required for efficient T-cell priming [15]. mDCs may play an important role in LASV infection, as both IFN-I and T-cell responses are important.

We previously showed that LASV replicated in monocyte-derived DCs (moDC) but did not activate them [10]. Here we further investigated the relevance of the escape of LASV from the DC response using another arenavirus, Mopeia virus (MOPV) [16]. MOPV is phylogenetically very close to LASV and has been isolated from *Mastomys natalensis*, the main reservoir of LASV [17]. However, MOPV is non-pathogenic in NHPs and can protect against LASV challenge [18]. No human case of MOPV infection has ever been reported [16]. Comparing MOPV and LASV, two very similar viruses but with different pathogenic potential, is a useful approach to identify immune and viral features involved in LASV pathogenesis. This comparison is used to model the differences between fatal and non-fatal LF *in vitro*. In moDCs, MOPV infection was productive and moderately activated cells [19]. In addition, MOPV-infected moDCs cultured with autologous T cells induced early T-cell activation, strong proliferation, and acquisition of effector, memory, and cytotoxic phenotypes. In contrast, LASV-infected moDCs only induced weak and delayed T-cell responses [20]. These results highlight the differences in the response of moDCs to MOPV and LASV, and their ability to induce T-cell responses. They further emphasize the link between DCs and the global response to LASV.

Nevertheless, recent findings have raised doubts concerning the validity of the moDC model. High dimensional mapping of the DC surface phenotype showed that moDCs do not match any *in vivo* population (DCs from blood, lymphoid organs, or skin) [21]. Thus, moDCs are not phenotypically representative of any DC subset in healthy individuals. Studies on influenza A virus also showed different susceptibility to infection between moDCs and primary mDCs [22]. In light of these results, we decided to investigate the role of mDCs during LASV infection using our LASV/MOPV comparison model.

We purified primary human mDCs and infected them with MOPV and LASV. mDCs infected with MOPV produced much higher levels of IFN-I than moDCs. Surprisingly, LASV-infected mDCs also produced high levels of IFN-I. Unlike moDCs, mDCs were not productively infected by MOPV or LASV, due to the strong IFN-I response. We further characterized the mDC response using transcriptomic and Luminex-based approaches. These large-scale results showed differences in the global activation of mDCs depending on the virus. We next tested the ability of mDCs to induce T-cell responses. Coculture with autologous T cells modulated the mDC response: MOPV-infected mDCs were more strongly activated when cultured with T cells, whereas activation of LASV-infected mDCs was inhibited. Furthermore, MOPV-infected mDCs induced early activation, proliferation, and cytotoxic responses of T cells. LASV-infected mDCs induced no or little T-cell activation. These results suggest crosstalk between mDCs and T cells, influencing not only T-cell responses but also mDC activation. We generated MOPV/LASV chimeras and tested them in our coculture model to identify viral parameters responsible for the differences between LASV and MOPV.

## Results

### mDCs are activated by MOPV and LASV infection

Previous studies showed that MOPV and LASV induced different IFN-I responses in moDCs [19,23]. We first investigated the kinetics of the IFN-I response to MOPV by mDCs. The synthesis of IFNα1, IFNα2, and IFNβ mRNAs after MOPV infection started as early as eight hours post-infection (hpi), peaked at 24 hpi, and then decreased to reach the level of that of uninfected cells at 40 hpi (S1A Fig). We detected no TNFα mRNA during MOPV infection. Based on these results, we quantified IFN-I at 24 hpi for the rest of the study.

**Fig 1:**
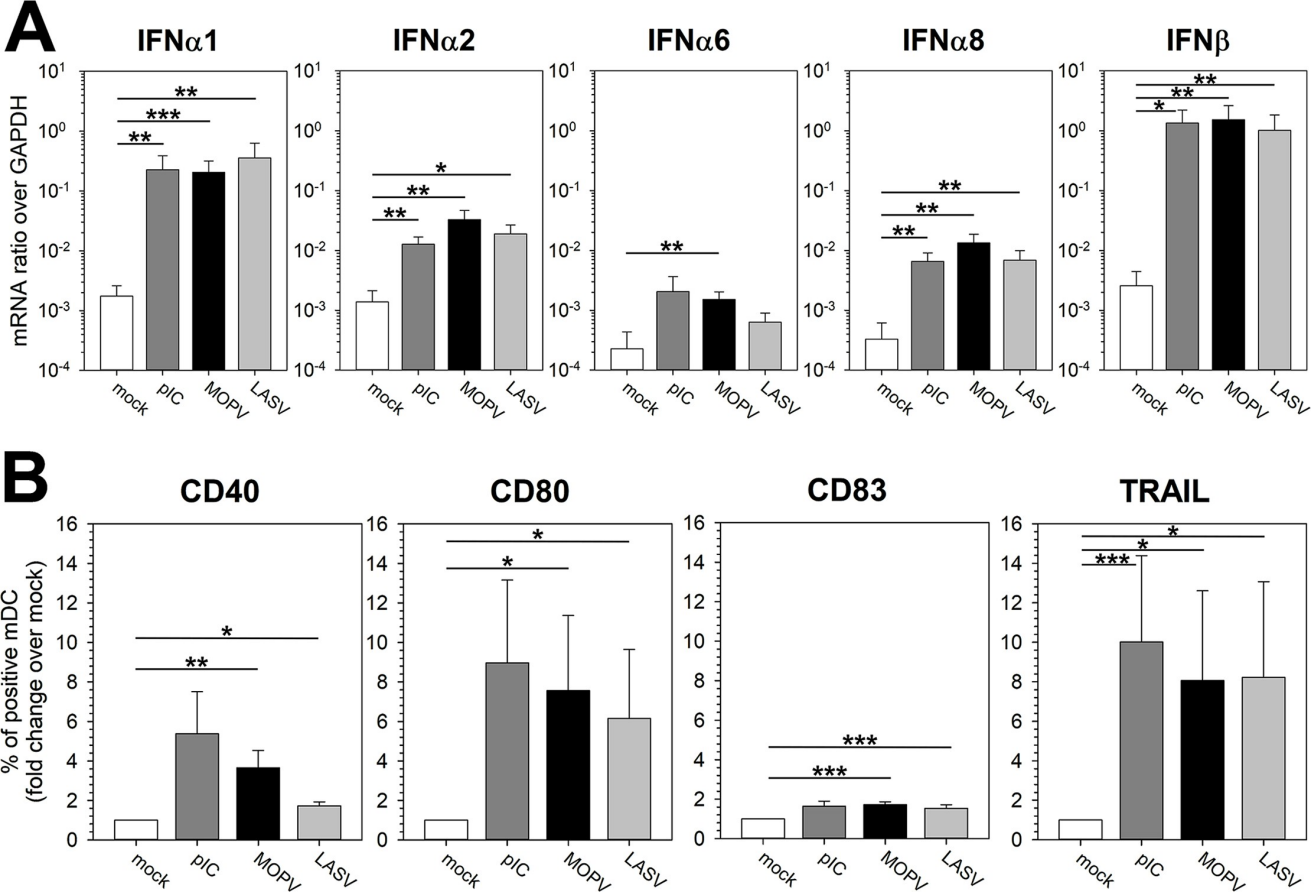
Primary human mDCs are activated by both MOPV and LASV. mDCs were cultured for 24 h with culture medium (mock), pIC (150 μg/mL), MOPV, or LASV (MOI = 2). Cells were analyzed by RT-qPCR (A) or flow cytometry (B). (A) Quantification of IFN-I mRNA is expressed as the gene/GAPDH ratio. (B) mDCs were gated as lineage^-^/HLADR^+^ and cells positive for activation molecules were counted. The results are expressed as the percentage of positive mDCs normalized over the mock condition. Data shown are the means and SEM of eight independent experiments. Statistical significance was assessed by the non-parametric Wilcoxon test and differences were considered to be significant for p < 0.05 (*), p < 0.01 (**), or p < 0.001 (***).

**Fig 2:**
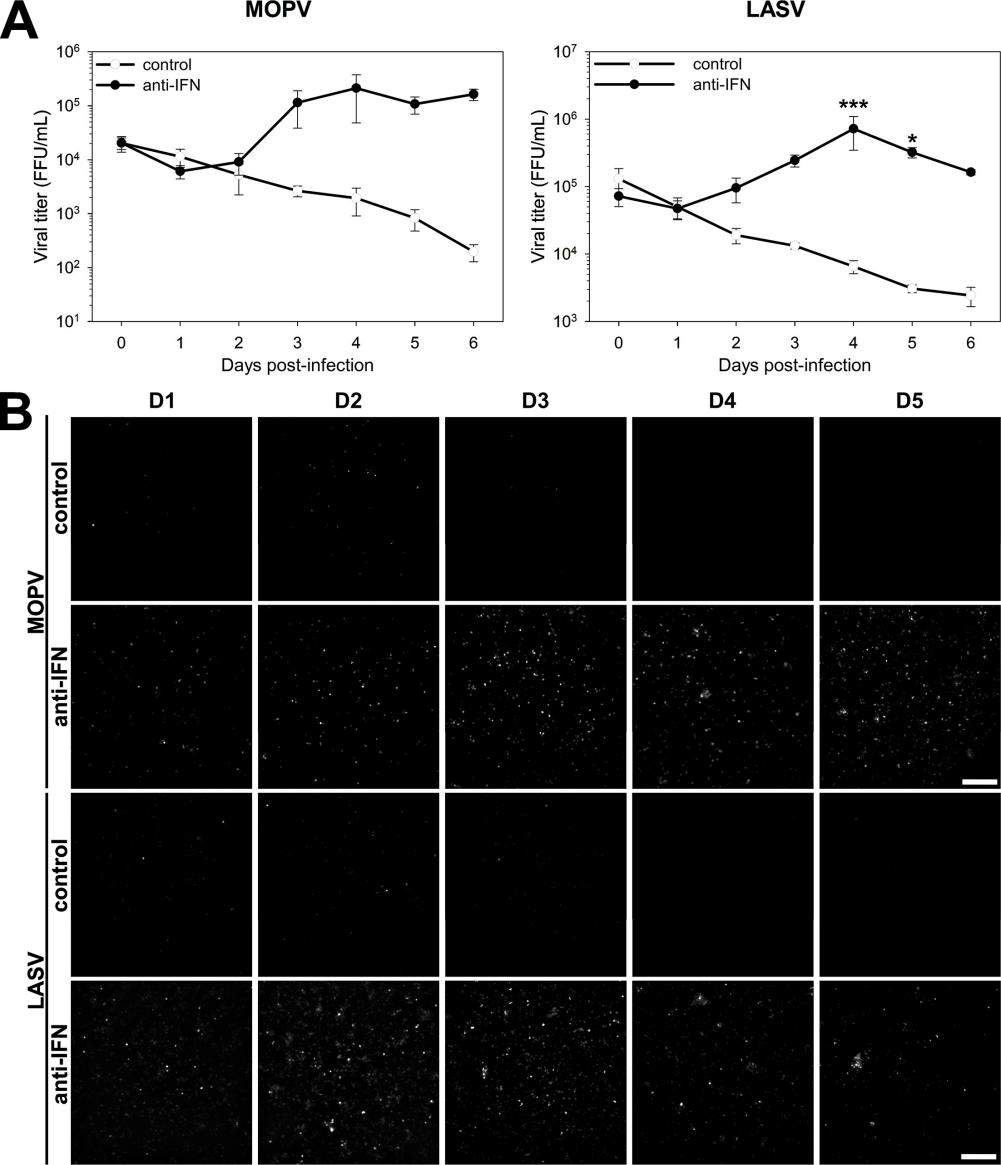
MOPV and LASV infection of mDCs is inhibited by the IFN-I response. mDCs were treated with cocktails of neutralizing antibodies 0, 1, and 3 days post-infection (dpi). In the anti-IFN condition, the cocktail contained antibodies neutralizing IFNAR (MMHAR-2, 5 μg/mL), IFNα (MMHA-2, 2.5 μg/mL), and IFNβ (MMHB-3, 2.5 μg/mL). In the control condition, the cocktail contained corresponding control isotype antibodies, IgG1 (5 μg/mL) and IgG2a (5 μg/mL). (A) mDCs were infected with MOPV or LASV (MOI = 0.1). Small volumes of supernatant were collected from 0 to 6 dpi and viral titers quantified. Data shown are the means and SEM of viral titers from three independent experiments. Statistical significance was assessed by Two-Way Repeated-Measures ANOVA followed by All Pairwise Multiple Comparison Procedures (Holm-Sidak method) and differences were considered to be significant for p < 0.05 (*), p < 0.01 (**), or p < 0.001 (***). (B) mDCs were infected with mCherry-expressing MOPV or LASV (MOI = 0.1). mCherry fluorescence was measured from 1 to 5 dpi. Images presented here are representative of three independent experiments.

The synthesis of IFNα1, IFNα2, and IFNβ, as well as that of IFNα6 and IFNα8 mRNA was significantly higher in MOPV-infected mDCs than in uninfected mDCs (Fig 1A). Surprisingly, LASV-infected mDCs also produced high levels of IFN-I, nearly similar to that of MOPV-infected mDCs. The IFN-I response to both viruses was associated with upregulation of the activation molecules CD40, CD80, CD83, and of TRAIL (Fig 1B). Viral titers in supernatants of MOPV- or LASV-infected mDCs decreased over time (Fig 2A, white circles), indicating that mDC infection by MOPV and LASV was not productive. Neither significant levels of transcription nor replication of either virus was detected in cells, as illustrated by the lack of mCherry synthesis in most cells after infection with mCherry-expressing LASV or MOPV (Fig 2B, control condition). However, we observed expression of mCherry in a few MOPV- and LASV-infected mDCs, indicating a low level of viral replication in a minority of mDCs during the two days following infection. We were also able to detect MOPV and LASV genomes both in the culture medium and in the cell pellet 24 hpi, with very low differences between MOPV and LASV, indicating that both viruses are able to enter into mDCs with a similar efficiency (S1B and S1C Fig). This was confirmed by the low level of MOPV and LASV tagged-Z protein expression detected in mDCs 2 days post-infection (dpi, S2C Fig). Together, these results suggest that there is no significant difference in the ability of LASV and MOPV to infect mDCs and in the lack of productive infection in these cells.

We investigated the possible relationship between the IFN-I response and the lack of viral replication. We thus inhibited the autocrine and paracrine effects of the IFN-I response with neutralizing antibodies that target the IFNα/β receptor (IFNAR), IFNα, and IFNβ. We treated mDCs with the anti-IFN cocktail and infected them with mCherry-expressing MOPV or LASV. mCherry was detected in the cells (Fig 2B, anti-IFN condition), indicating that both viruses replicated. We confirmed this result by titrating infectious particles in the culture medium. Under the anti-IFN condition, viral titers increased from one to four dpi (Fig 2A, black circles), with curves similar to those of classical replication kinetics of MOPV and LASV. Therefore, neutralizing the IFN-I response was sufficient to establish productive infection in mDCs. We demonstrated that mDCs are *per se* permissive to MOPV and LASV infection, but infection is inhibited by the early and robust IFN-I response.

### Global state of activation in MOPV- and LASV-infected mDCs

Our study of mDC responses to MOPV and LASV showed, so far, no significant differences between the two viruses. We thus used large-scale methods to obtain a deeper insight into these responses.

**Fig 3:**
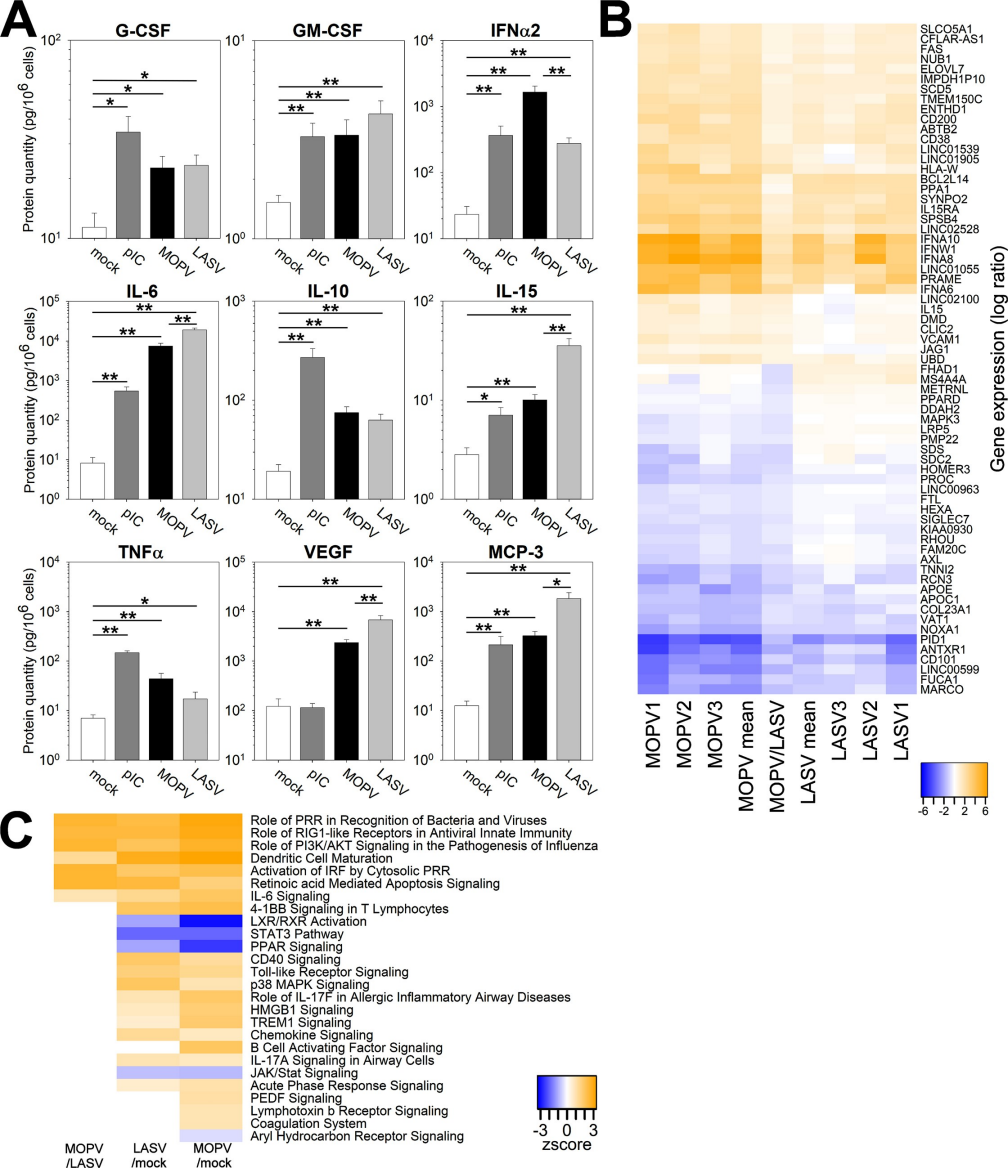
Activation patterns are different between MOPV- and LASV-infected mDCs. (A) mDCs were cultured for 24 h with culture medium (mock), pIC (150 μg/mL), MOPV, or LASV (MOI = 2). Proteins levels were quantified in the culture medium using the Luminex assay. Data shown are the means and SEM of five independent experiments. Statistical significance was assessed by the non-parametric Wilcoxon test and differences were considered to be significant for p < 0.05 (*) or p < 0.01 (**). (B-C) mDCs were cultured for 24 h with culture medium (mock), MOPV, or LASV (MOI = 1). Cellular mRNA from three independent experiments was quantified using poly-A amplification and next-generation sequencing. (B) The central column shows differential expression of genes in MOPV relative to LASV (MOPV/LASV). Other data shown are the differential expression of genes in the MOPV and LASV condition relative to mock for independent experiments (1, 2, and 3) or the mean of three experiments (mean). Genes shown in this figure displayed significant differences of expression between the MOPV- and LASV-infected conditions (adjusted p < 0.05). (C) Regulation of pathways between mock, MOPV, and LASV conditions was analyzed using Ingenuity Pathway Analysis. Z-scores were computed from the differential expression of significantly regulated genes (adjusted p < 0.05): in the MOPV/mock column, a positive Z-score indicates that the pathway is globally upregulated in MOPV-infected mDCs relative to uninfected mDCs.

We first focused on molecules released by mDCs in the culture medium using the Luminex assay (Fig 3A). There was significantly more IFNα2 in the supernatants of MOPV-infected mDCs than those of LASV-infected mDCs. Chemokines (MCP-1, MCP-3, and IP-10) and growth factors (VEGF, G-CSF, and GM-CSF) were produced by MOPV- and LASV-infected mDCs. Many pro-inflammatory cytokines were also upregulated in both MOPV- and LASV-infected mDCs: IL-5, IL-6, IL-10, IL-12, IL-15, and TNFα. In our study, IL-6, IL-15, VEGF, and MCP-3 were particularly interesting, because they were produced in higher quantities by LASV-infected than MOPV-infected mDCs.

Next, we used a transcriptomic approach to obtain an overview of mDC gene transcription. The differential expression of genes in MOPV- or LASV-infected mDCs relative to uninfected mDCs is presented in Fig 3B. The central column (MOPV/LASV) corresponds to the differential expression of genes between MOPV-infected mDCs and LASV-infected mDCs. The plotted genes show significant differences in expression between MOPV-infected mDCs and LASV-infected mDCs. Globally, genes up (or down) regulated in MOPV-infected mDCs relative to mock-infected mDCs were also up (or down) regulated in LASV-infected mDCs. However, the fold change of expression was generally higher for MOPV than LASV. Thus, the genes upregulated by MOPV were statistically more highly induced than those by LASV. As expected, many genes differentially regulated between MOPV- and LASV-infected mDCs were related to the immune response: IFN (IFNα6, IFNα8, IFNα10, and IFNω1), pro-inflammatory (IL15, IL15RA, SIGLEC7, and AXL), and cell-cell communication (CD101, CD200, HLA-W, MS4A4A, and HOMER3). We also identified genes involved in cell growth and death (Fas, BCL2L14, PRAME, and PID1), adhesion and mobility (SYNPO2, DMD, COL23A1, and VCAM1), or both (ANTXR1, AXL, and SDC2). More surprisingly, metabolic genes were regulated, especially those for lipid metabolism (ELOVL7, SCD5, METRNL, LRP5, APOE, APOC1, and FUCA1), oxidative molecule regulation (SPSB4, DDAH2, and NOXA1), and ionic transport (SLCO5A1, TMEM150C, CLIC2, FAM20C, and TNNI2).

These differences had an important effect on the global activation state. Indeed, various pathways were regulated in LASV- and MOPV-infected mDCs relative to mock-infected mDCs (Fig 3C). Most of them were related to the immune response, showing that mDCs responded to both viruses. However, there were substantial differences between MOPV- and LASV-infected mDCs at the pathway level. Immunity-related pathways, such as Pattern Recognition Receptors or RIG-I Like Receptor signaling, were upregulated in MOPV-infected mDCs relative to LASV-infected mDCs (Fig 3C). DC maturation was also upregulated by MOPV relative to LASV. This difference was mostly caused by the upregulation of IFN-I, CD40, CD83, CCR7, IL-6, NFκB (p50), MDA5, and Fas genes in MOPV-infected mDCs, but not LASV-infected mDCs (S1D Fig). Overall, these results highlight differences in the activation state of MOPV- and LASV-infected mDCs.

### MOPV-infected mDCs, but not LASV-infected mDCs, activate T cells

DCs are involved in the innate immune response. However, a major role of mDCs *in vivo* is to induce a specific cellular response. We developed a coculture model with mDCs (infected or not) and autologous T cells (CD4 and CD8). In these cocultures, the amount of MOPV and LASV genomes in the culture medium and in the cells decreased over time, suggesting an absence of viral replication (S2A and S2B Fig). To verify this hypothesis, we stained purified mDCs, purified T cells and mDC-T cocultures for intracellular Z protein. We found no positive T cells (S3 Fig), indicating that viral particles do not enter CD4 and CD8 T cells. mDCs infected by MOPV and LASV had a detectable level of intracellular Z, but only when cultured without T cells (S2C Fig). Considering the low intensity of the Z staining compared to permissive A549 cells, the detected Z proteins more likely come from internalized particles than from new virions.

**Fig 4:**
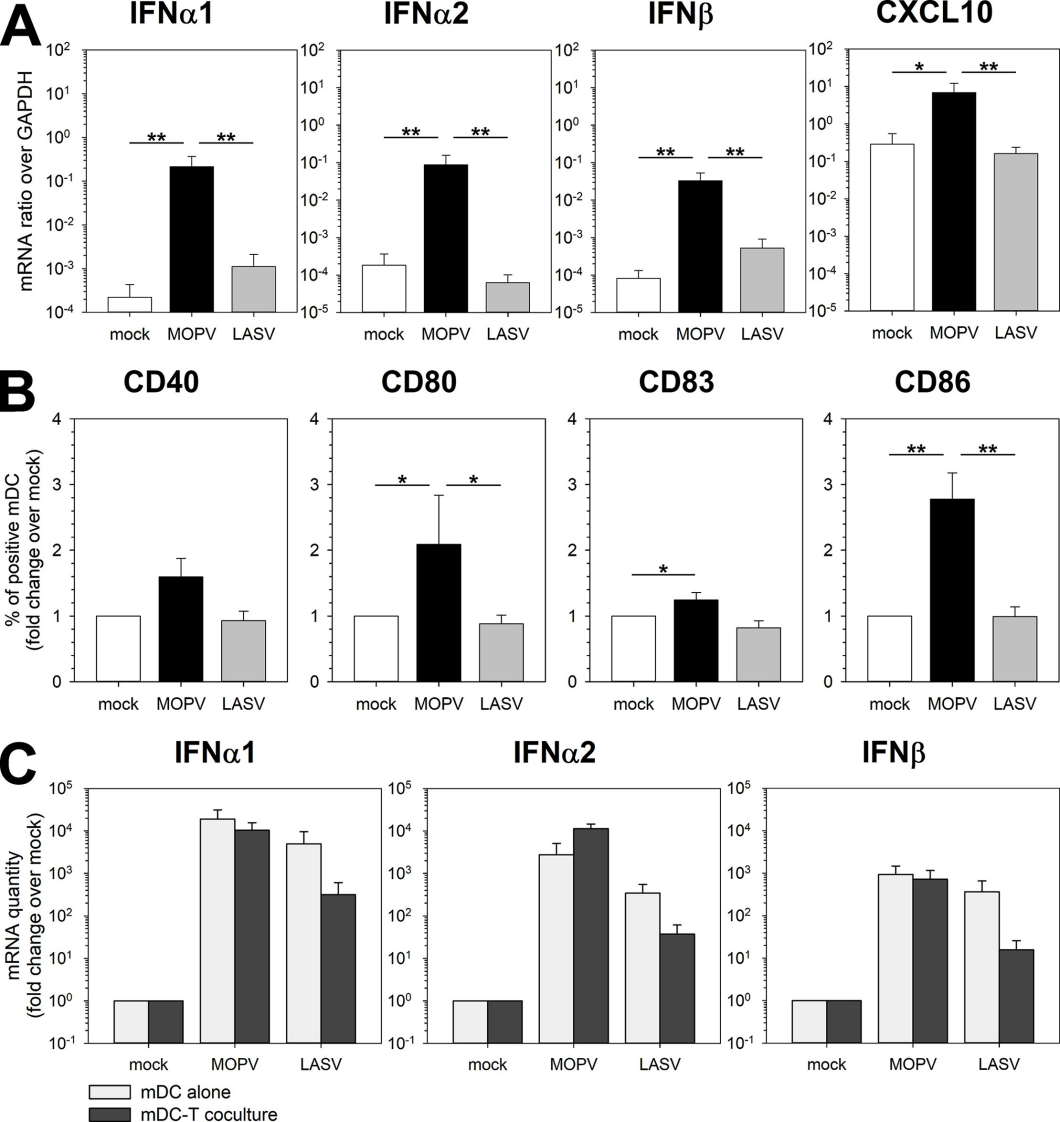
The mDC response to MOPV and LASV is modulated by coculture with T cells. mDCs were infected with MOPV or LASV or were uninfected and cultured for 24 (A-C) or 48 h (B) with T cells. mDCs were analyzed by RT-qPCR (A-C) or flow cytometry (B). (A) Quantification of IFN-I and CXCL10 mRNA is expressed as the gene/GAPDH ratio. (B) mDCs were gated as lineage^-^/HLADR^+^, and cells positive for activation molecules counted. Results are expressed as the percent of positive mDCs normalized over the mock condition. Data shown are the means and SEM of seven independent experiments. Statistical significance was assessed by the non-parametric Wilcoxon test and differences were considered to be significant for p < 0.05 (*) or p < 0.01 (**). (C) Results of the IFN-I response in mDC-T cell coculture (presented in Fig 4A) compared to those for infected mDCs (from Fig 1A). mDCs represented only 10% of total cells expressing GAPDH in mDC-T cell cocultures, but were the main cell type expressing IFN-I.

We then investigated the response of mDCs using this coculture model. MOPV induced substantial IFN-I and CXCL10 production by mDCs at 24 hpi, whereas LASV did not (Fig 4A). At 2 dpi, IFN-I and CXCL10 were upregulated under both MOPV and LASV conditions (S4A Fig). MOPV-infected mDCs upregulated the activation markers CD40, CD80, CD83, and CD86 (48 hpi), but LASV-infected mDCs did not (Fig 4B). These results were surprising, as they did not match those obtained with infected mDCs alone (Fig 1). Fig 4C shows the IFN-I response at 24 hpi for MOPV- and LASV-infected mDCs (mDC alone, results from Fig 1A) and mDCs in coculture (mDC-T coculture, results from Fig 4A). Under uninfected conditions, IFN-I mRNA levels were 10-fold lower in coculture than when mDCs were cultured alone. This difference was expected because mRNA levels were normalized to those of GAPDH (present in all cells) and IFN-I is mostly produced by mDCs (10% of the cells). MOPV infection of mDCs induced a 10- (IFNα1) to 80- (IFNα2) fold increase in IFN-I production relative to that of mock-infected cells when cocultured with T cells. Remarkably, coculture with T cells completely inhibited IFN-I synthesis by LASV-infected mDCs: alone, IFN-I production was comparable to that of MOPV-infected mDCs, and in coculture it was comparable to that of the mock condition. Altogether, these results show that mDC activation and the IFN-I response to MOPV were increased and prolonged in coculture, whereas activation and the IFN-I response to LASV were reduced and delayed. This suggests crosstalk between mDCs and T cells, which modulate the mDC response to MOPV and LASV.

**Fig 5:**
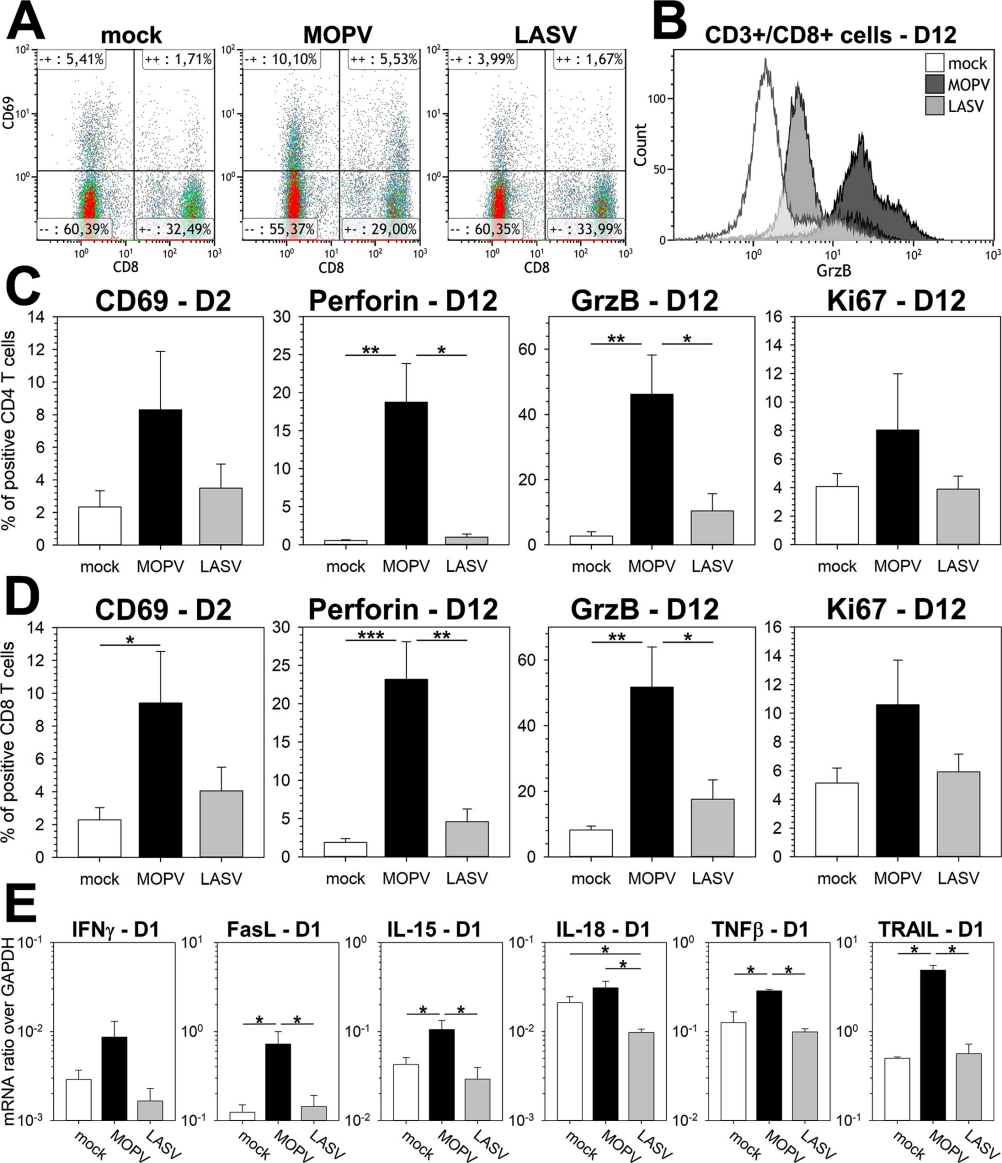
MOPV-infected mDCs, but not LASV-infected mDCs, induce T-cell activation and a cytotoxic response. mDCs were infected with MOPV or LASV (MOI = 1) or were uninfected and cultured with T cells for 1, 2, or 12 days (see indications on graphs). T cells were analyzed by flow cytometry (A-B-C-D) or RT-qPCR (E). (A) Dot plot showing expression of CD69 in CD3^+^ cells 2 dpi. CD8^+^ cells correspond to CD8 T cells and CD8^-^ cells to CD4 T cells. (B) Distribution of GrzB expression in CD8 T cells (CD3^+^/CD8^+^) 12 dpi. Data shown are of one experiment representative of seven replicates. (C-D) CD4 T cells were gated as CD3^+^/CD4^+^ cells (C) and CD8 T cells as CD3^+^/CD8^+^ cells (D). Cells positive for activation molecules were counted. Results are expressed as the percentage of positive CD4 (C) or CD8 (D) T cells. Data shown are the means and SEM of seven independent experiments. (E) Quantification of mRNA is expressed as the gene/GAPDH ratio. Data shown are means and SEM of four independent experiments. Statistical significance was assessed by the non-parametric Wilcoxon test and differences were considered to be significant for p < 0.05 (*) or p < 0.01 (**).

Lastly, we studied T cell responses in our model using flow cytometry. We quantified CD69, a marker of early T cell activation, 2 dpi (Fig 5A). CD69 was upregulated in both CD4 (Fig 5B) and CD8 (Fig 5C) T cells under conditions of MOPV infection, but not those of LASV infection. By 12 dpi, CD4 and CD8 T cells cultured with MOPV-infected mDCs expressed higher amounts of the cytotoxic molecules perforin and granzyme B (GrzB, Fig 5B–5D). Such a cytotoxic phenotype was not induced by LASV-infected mDCs. At 15 dpi, there was slight upregulation of perforin and GrzB after coculture with LASV-infected mDCs, but it was still lower than that for the coculture with MOPV-infected mDCs (S4B and S4C Fig). Ki67 expression by CD4 and CD8 T cells was also upregulated by MOPV-infected mDCs, suggesting their proliferation. Therefore, MOPV-infected mDCs induced activation, a cytotoxic response, and proliferation of CD4 and CD8 T cells. LASV-infected mDCs poorly activated T cells. We searched for factors that could influence the mDC response at early time points. The MOPV coculture was characterized by upregulation of IFNγ, Fas ligand (FasL), IL-15, TNFβ, and TRAIL (Fig 5E). None of these molecules were upregulated in the LASV coculture, and they may play a role in the modulation of mDC responses. Interestingly, IL-18 was downregulated under conditions of LASV infection relative to those of mock or MOPV infection.

### The major immunosuppressive properties of LASV are carried by the NP

**Fig 6:**
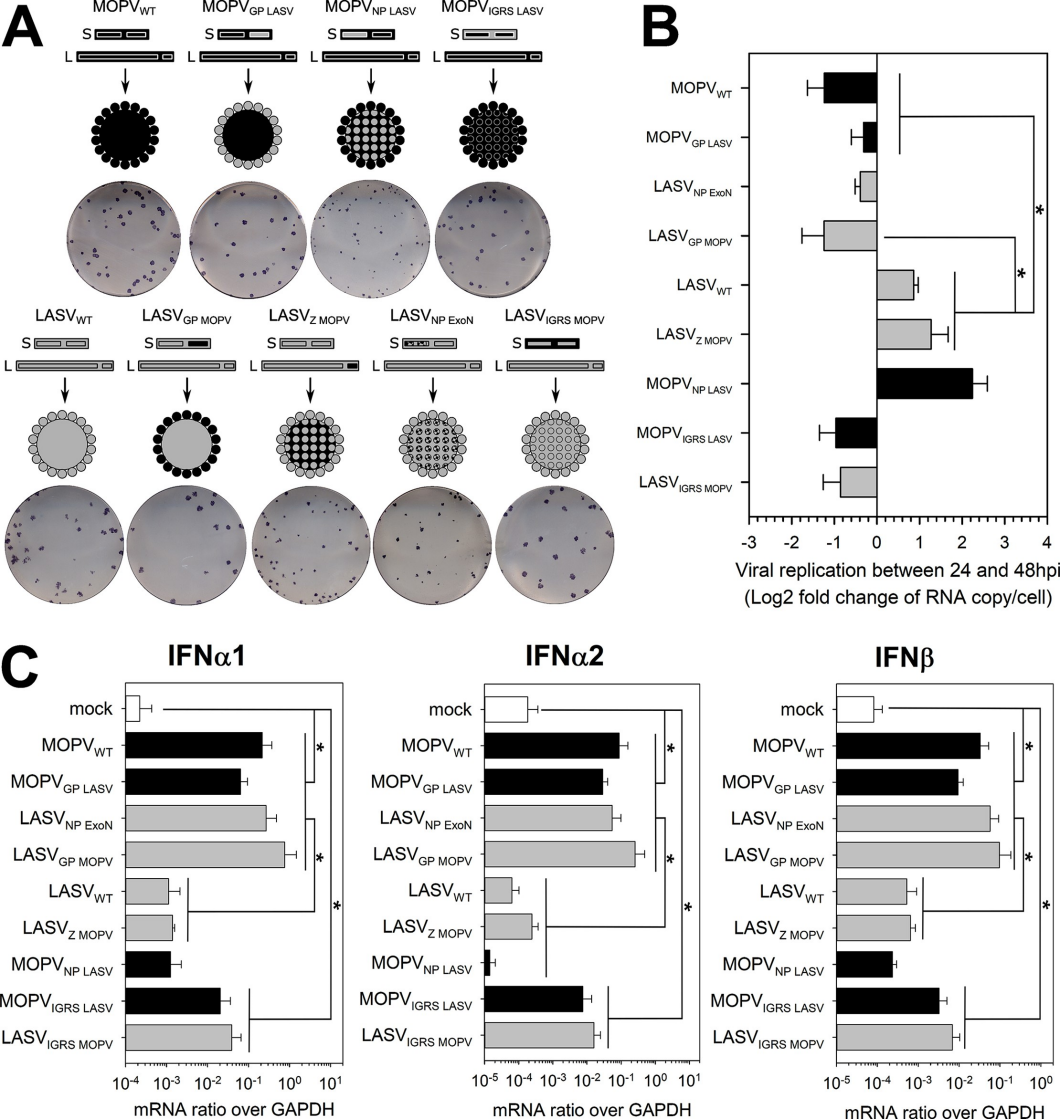
Exchanging viral proteins between MOPV and LASV modifies mDC activation in the context of mDC-T-cell coculture. (A) Generation of chimeric MOPV and LASV viruses. In the schematic views, the black segments correspond to MOPV elements and grey segments to LASV elements. LASV_NP ExoN_ has mutations in the NP exonuclease domain that make it nonfunctional. MOPV_IGRS LASV_ and LASV_IGRS MOPV_ have exchanged intergenic regions of the S segment. The images show the plaque phenotypes of the viruses. (B-C) mDCs were infected with wild type (WT) or chimeric viruses (MOI = 1) or were uninfected and cultured with T cells for one or two days. (B) Fold change between 1 and 2 dpi in intracellular viral RNA levels (log2([RNA copy per cell at 2 dpi]/[RNA copy per cell at 1 dpi]). (C) Quantification of IFN-I mRNA at 1 dpi is expressed as the gene/GAPDH ratio. Black and grey bars correspond to viruses with the MOPV and LASV backbones, respectively. Data shown are the means and SEM of four independent experiments. Statistical significance was assessed by the non-parametric Wilcoxon test and differences were considered to be significant for p < 0.05 (*).

After having investigated the differences between the responses of MOPV- and LASV-infected mDCs in coculture, we assessed the role played by viral factors. We designed MOPV/LASV chimeras by swapping the different viral proteins between the viral backbones (Fig 6A). We obtained MOPV in which its GP, NP, or intergenic regions of the S segment (IGRS) were replaced by their LASV counterparts, and the corresponding LASV containing MOPV GP, Z, or IGRS. Successful exchanges of the ORFs were verified by next generation sequencing (S5 Fig) and western blot (S6A Fig). We also included LASV_NP ExoN_, an LASV mutant with a non-functional exonuclease domain in its NP (described in [24,25]). As expected, some chimeric viruses displayed a reduced and delayed growth in VeroE6 cells compared to wild type viruses (S6B Fig). The genomes/infectious particles ratio varied between viral stocks, but with the exception of LASV_NP ExoN_, differences were rather low (S6B and S6C Fig).

We tested the chimeras in our mDC-T coculture model. Quantification of the IFN-I response allowed us to segregate the viruses into three groups: MOPV-like, LASV-like, and intermediate. In the MOPV-like group (MOPV_WT_, MOPV_GP LASV_, LASV_NP ExoN_ and LASV_GP MOPV_), infected mDCs produced large amounts of IFN-I mRNA. In the LASV-like group (LASV_WT_, LASV_Z MOPV_ and MOPV_NP LASV_), the IFN-I response was as low as under the uninfected condition. MOPV_IGRS LASV_- and LASV_IGRS MOPV_-infected mDCs produced small amounts of IFN-I and were designated as “intermediate”. It is worth noticing that mDC activation by the chimeric viruses in mDC-T coculture did not match their attenuation on VeroE6 cells: LASV_NP ExoN_ and MOPV_NP LASV_ were the most attenuated ones, whereas their phenotypes in mDC-T coculture were completely different. These results confirmed that the differences we observed in the responses are due to the activity of viral proteins and not to defects in viral replication and assembly. The viral titers did not detectably increase over time for any of the viruses, even though LASV_WT_ titer seemed to decrease more slowly (S6D Fig). Quantification of intracellular viral RNA at 1 and 2 dpi (Fig 6B) showed an increase in viral RNA for only LASV-like viruses. Inhibition of the IFN-I response appeared to be associated with viral replication, even at low levels, without viral particle release.

**Fig 7:**
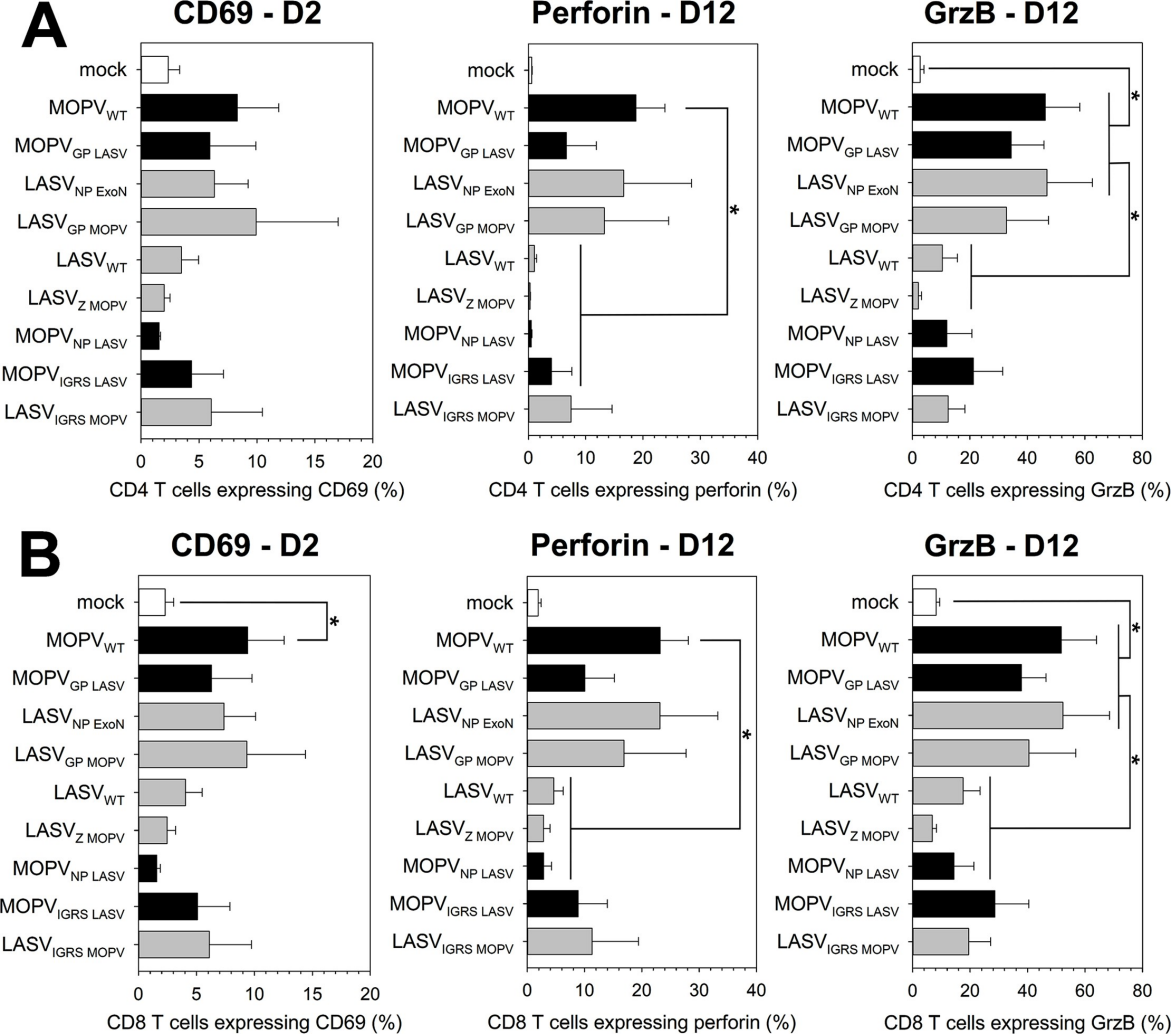
T cell responses to chimeric viruses correlate with mDC activation. mDCs were infected with WT or chimeric viruses (MOI = 1) or were uninfected and cultured with T cells for 1, 2, or 12 days (see indications on graphs). T cells were analyzed by flow cytometry. CD4 T cells were gated as CD3^+^/CD4^+^ cells (A) and CD8 T cells as CD3^+^/CD8^+^ cells (B). Cells positive for activation molecules were counted. Results are expressed as the percentage of positive CD4 (A) or CD8 (B) T cells. Black and grey bars correspond to viruses with the MOPV and LASV backbones, respectively. Data shown are the means and SEM of four independent experiments. Statistical significance was assessed by the non-parametric Wilcoxon test and differences were considered to be significant for p < 0.05 (*).

Differences identified in the mDC response were also observed in the T-cell response (Fig 7). MOPV-like viruses induced over-expression of CD69, perforin, and GrzB in CD4 (Fig 7A) and CD8 (Fig 7B) T cells. LASV-like viruses did not induce T-cell activation. The intermediate viruses showed low-level overexpression of CD69, perforin, and GrzB. The T-cell response correlated with the IFN-I response of mDCs in coculture.

This approach provided useful information on the immunogenic/immunosuppressive properties of viral proteins. MOPV Z protein did not alter the ability of LASV to escape from or suppress mDC and T-cell responses. Exchanging the IGRS between LASV and MOPV partially reversed their phenotype, suggesting that MOPV IGRS is more immunogenic than LASV IGRS. However, the immunogenicity of IGRS was not sufficient to explain the differences between MOPV_WT_ and LASV_WT_. LASV GP did not appear to have immunosuppressive properties, as MOPV_GP LASV_ exhibited a MOPV-like phenotype. In contrast, MOPV GP was sufficient to render LASV as immunogenic as MOPV. This immunogenicity was associated with the lack of viral replication of LASV_GP MOPV_ (Fig 6B). The most striking result of this experiment was the role of the NP. MOPV_NP LASV_ behaved as LASV_WT_, showing that LASV NP was sufficient to completely abolish MOPV immunogenicity. The fact that LASV_NP ExoN_ was as immunogenic as MOPV suggests that the immunosuppressive properties of LASV NP require a functional exonuclease domain.

In summary, we showed that the inhibition of mDC and T-cell activation by LASV requires the expression of a functional NP with exonuclease properties. Our results also suggest that MOPV GP affects the ability of LASV to replicate in cells, influencing its immunogenicity. Finally, MOPV and LASV IGRS had different immunogenic properties.

## Discussion

We investigated the immune response parameters involved in the control of LASV infection or possibly associated with severe disease and death by studying the response of primary human mDCs to LASV and MOPV. We used MOPV infection to model non-fatal LF, as MOPV is very closely related to LASV, but non-pathogenic in humans [10,19,20,23]. We showed that mDCs were activated, acquired a mature phenotype (CD83 expression), and produced high levels of IFN-I mRNA in response to both MOPV and LASV infection. mDC activation was associated with detection of viral genomes on and/or inside the cells, but not with a significant productive infection. These results were surprising, as MOPV is much more immunogenic than LASV. Previous studies showed that moDCs, which are often used as a model for mDCs, produced moderate amounts of IFN-I in response to MOPV, but not LASV infection [10,19]. In addition, moDCs were productively infected by MOPV and LASV, whereas mDCs were not. This difference is consistent with the fact that LASV replication in LPS-matured moDC was lower than that in immature cells [10]. We also demonstrated that the IFN-I response of mDCs to both viruses was sufficient to explain the absence of productive infection.

We then conducted a large-scale study to exhaustively assess mDC responses to MOPV and LASV. We identified cytokines produced by MOPV- and LASV-infected mDCs, most which are found in patients with terminal VHF [26]. Five of these proteins were produced in different amounts by MOPV-infected and LASV-infected mDCs: IFNα2, IL-6, IL-15, VEGF, and MCP-3. IFNα2 levels were significantly higher in MOPV-infected mDCs than LASV-infected cells. This result was consistent with the non-significant differences observed in IFNα2, IFNα6, and IFNα8 mRNA levels between MOPV- and LASV-infected mDCs. The other four proteins were produced in higher amounts during LASV infection. IL-6 is known to be associated with severe cases of VHF [27] and IL-15 production has been correlated with severe cases in CCHF patients [28]. VEGF has been associated with plasma leakage and severity in dengue hemorrhagic fever [29], and MCP-3 has recently been described in Ebola virus-infected NHPs [30]. These factors could all have a role in the pathogenesis of LF, even though DCs are not necessarily the main cell type producing them *in vivo*.

MOPV infection strongly altered the transcriptomic state of mDCs. Most of the genes up (or down) regulated during MOPV infection were also up (or down) regulated during LASV infection, but at much lower levels. Many genes differentially regulated between MOPV- and LASV-infected mDCs were linked to immunity. We also identified genes involved in cell mobility and survival and, more surprisingly, ion and lipid metabolism. The relationship between lipids and VHF has been highlighted in other studies: lipids in serum are associated with febrile illness in LF patients [31] and the LXR/RXR pathway, which integrates lipid metabolism and immune functions, is necessary for dengue virus replication [32]. These differentially regulated genes had an impact on a larger scale: pathways linked to the innate immune response, such as PRR recognition, RIG-I like receptors, PI3K signaling, and DC maturation, were significantly upregulated in MOPV-mDCs relative to LASV-infected mDCs. The regulation of these pathways mostly relied on the same set of genes, including IFN-I, NFκB, CD40, and CD80. Such pathway analysis showed that regulated genes had an important effect on the global activation state of the cells. The main observation was that immunity-related genes and pathways were more highly up-regulated during MOPV infection than LASV infection. Altogether, the differences in transcriptomic regulation confirmed the higher immunogenicity of MOPV than LASV.

*In vivo*, the ability of mDCs to induce specific T-cell responses could be critical during LASV infection. Indeed, the control of LF seems to rely mostly on robust T-cell responses [9,33]. We developed a mDC/T cell coculture system to measure the immunogenicity of MOPV- and LASV-infected mDCs through their ability to induce specific T-cell responses. When cultured with T cells, neither MOPV nor LASV infection seemed to be productive. However, the presence of T cells affected the amount of Z detected inside mDCs 2 to 8 dpi. Our hypothesis is that interaction with T cells accelerated the recycling of major histocompatibility complexes, and therefore the degradation of viral antigens. In this system, MOPV-infected mDCs produced even higher amounts of IFN-I than alone and induced T cell activation, proliferation, and acquisition of a cytotoxic phenotype. On the contrary, LASV-infected mDCs were not activated, did not produce IFN-I, and poorly activated T cells. These differences in T-cell activation are similar to those observed for moDCs. However, mDCs appeared to be much more immunogenic than moDCs: a single stimulation with mDCs was sufficient to activate T cells, whereas multiple stimulation with moDCs were required [20]. Therefore, moDCs are a relevant model to study T-cell activation in the context of MOPV and LASV infection, but not the innate immune response. In this study, T cells primed with MOPV-infected moDCs acquired the ability to specifically release GrzB in the presence of MOPV-infected cells, controlling viral replication [20]. Although we were unable to test this in our model, T cells expressing GrzB and perforin probably had the ability to lyse MOPV-infected cells. Indeed, naïve T cells do not contain cytotoxic granules. GrzB and perforin expression only occur after TCR stimulation and T-cell proliferation [34,35]. T cells primed with MOPV-infected mDCs expressed GrzB and perforin and were thus most likely fully functional cytotoxic T cells. The robust and efficient T-cell responses induced by MOPV-infected mDCs may result from the strong observed transcriptional activation of mDCs. In contrast, LASV-infected mDCs showed lower, but significant, activation and failed to induce the activation and proliferation of T cells. These observations correlate with the high immunogenicity and non-pathogenicity of MOPV, and low immunogenicity and high pathogenicity of LASV.

T cells potentiated the activation of MOPV-infected mDCs, resulting in a 10-fold increase in IFNα release relative to that of mDCs alone. On the contrary, T cells had an inhibitory effect on LASV-infected mDCs, abrogating the synthesis of IFN-I mRNA and the expression of co-stimulatory molecules observed in LASV-infected mDCs. This suggests early crosstalk between mDCs and T cells, resulting in bilateral activation during MOPV-infection and mDC suppression during LASV-infection. The crosstalk between mDCs and T cells could occur through direct contact or soluble mediators. IFNγ, IL-15, TNFβ, FasL, and TRAIL mRNA were overexpressed in the presence of MOPV-infected cells relative to uninfected and LASV-infected cells. These molecules, mainly released by activated T cells, may have been involved in the strengthening of mDC and T cell activation. However, NK cells could have participated in the release of IFNγ and TRAIL, as these cells were still present in the cultures. In the presence of LASV, the level of these T cell-derived mRNAs did not increase. Levels of IL-18, an APC-derived cytokine, were even significantly lower, confirming the inhibition of the mDC response to LASV. Further studies are needed to identify the mechanism involved in this negative activation loop between mDCs and T cells.

We generated MOPV/LASV chimeras to identify viral parameters responsible for the differences in T cell activation by MOPV- and LASV-infected mDCs. MOPV_GP LASV_, LASV_NP ExoN_, and LASV_GP MOPV_ showed similar immunogenicity as MOPV_WT_. LASV_Z MOPV_ and MOPV_NP LASV_ were as poorly immunogenic as LASV_WT_. MOPV_IGRS LASV_ and LASV_IGRS MOPV_ displayed an “intermediate” phenotype. The segregation of viruses in these three phenotypes did not correlate with their attenuation on VeroE6, cells, so the activation of mDC-T cocultures by the chimeras cannot be explained by assembly defects. The “LASV-like” viruses were the only ones that showed productive infection. The low activation of mDCs and limited production of IFN-I probably allowed some viral replication, which was still quickly controlled 2 dpi when IFN-I was synthesized. The impact of the IGRS on immunogenicity presumably relied on a difference of structure: the MOPV IGRS has a duplicated hairpin structure, whereas that of LASV has only one [36]. The double hairpin is most likely a better stimulator of intracellular sensors than a single hairpin, and thus more immunogenic. This could explain the intermediate phenotype of IGRS chimeras. Nevertheless, this difference between their IGRS was not sufficient to explain the differences in immunogenicity between MOPV_WT_ and LASV_WT_, confirming that recognition by intracellular sensors is not the only mechanism involved. We also showed that MOPV Z protein was not a modulator of immunogenicity. We were unable to test LASV Z properties in our model, as MOPV_Z LASV_ was not viable. However, the removal of LASV Z did not affect LASV immunogenicity, indicating that it does not have major immunosuppressive properties. Previous studies suggested that the Z protein of some arenaviruses is able to bind RIG-I and inhibit its signaling, resulting in an inhibition of IFN-I response. One of these studies showed that the Z proteins of New World arenaviruses, but not Old World arenaviruses, inhibit RIG-I signaling, which is coherent with our findings [37]. A more recent study concluded that only the Z proteins of pathogenic arenaviruses, including Old World arenaviruses, present this immunosuppressive function, suggesting that LASV Z (but not MOPV Z) is immunosuppressive [38]. However, we did not find in our mDC model any evidence for an immunosuppressive role of either LASV or MOPV Z protein. These discrepancies could be due to the use of different models. Indeed, additional sensors other than RIG-I could contribute for type I IFN synthesis in response to arenavirus infection in human mDC and therefore could conceal the effect of LASV Z protein on RIG-I. MDA5 would be a possible candidate for such an hypothesis. LASV GP did not appear to have major immunosuppressive properties. Indeed, MOPV_GP LASV_ was almost as immunogenic as MOPV and did not replicate. In contrast, LASV became as immunogenic as MOPV when it expressed MOPV GP. It is possible that MOPV and LASV GP address virions to different cellular compartments, influencing their ability to escape or trigger innate immunity and, consequently, viral replication. Their possible use of different fusion receptors, Lamp1 for LASV and unidentified for MOPV, may also be involved [39]. We are currently investigating this possibility. Finally, our results showed that LASV NP was sufficient to abolish the immunogenicity of MOPV-infected mDCs. This shows that LASV NP has major immunosuppressive properties, which require its exonuclease domain. The immunosuppressive properties of arenavirus NP, especially of its exonuclease domain, have already been largely described. This domain is required for LASV NP to inhibit the IRF pathway [40]. LASV detection by RIG-I is inhibited through the digestion of double-stranded RNA by LASV NP, and this mechanism is probably conserved among most arenaviruses [25,41,42]. However, the discrepancy between the ability of MOPV and LASV to modulate immunogenicity is surprising, as both NPs contain an active exonuclease domain [43]. It is possible that the exonuclease activity of LASV NP is more potent than that of MOPV. Another possibility is that the digestion of dsRNA is not the only function of this domain. The ability of LASV NP to bind IKKε may be involved, as this interaction is associated with the exonuclease domain [44]. LASV NP also interacts with the PACT complex, inhibiting PACT-dependent activation of RIG-I [45]. This interaction is abolished when the exonuclease domain is mutated. NP-PACT interaction has been shown for Junin, Machupo, Tacaribe and Pichinde, but MOPV was not included in this study. Other cellular proteins interacting with LASV NP have been identified, but not yet described, and could be involved in its immunosuppressive properties [46]. Unfortunately, it was not possible to study the impact of MOPV NP on LASV immunogenicity, because LASV_NP MOPV_ was not viable.

In conclusion, this *ex vivo* approach showed that primary human mDCs respond well to both MOPV and LASV. However, only MOPV-infected mDCs were activated and able to induce T-cell responses when cultured with T cells. We demonstrated that NP and GP are viral factors involved in the respective immunogenic and immunosuppressive properties of MOPV and LASV. Duplicated hairpins in the S segment, different cell addressing by GP, and less efficient immunosuppressive factors, such as NP, may result in efficient triggering of RNA sensors by MOPV, explaining its strong immunogenicity. In contrast, multiple and efficient immunosuppressive mechanisms, mediated by NP and Z, and a lower ability of viral RNA to stimulate cell receptors are probably responsible for the poor immunogenicity of LASV. The comparison of LASV and MOPV is used to model the differences between fatal and non-fatal LF *in vitro*. *In vivo*, the factors that determine the outcome of LF are still unknown. Factors involved in different mDC responses are good candidates, as mDCs may initiate the entire immune response in humans. Host polymorphisms, initial viral load, and the route of infection may affect LASV replication in mDCs, the detection of LASV by intracellular sensors, and the inhibition of mDC activation by viral proteins. Such factors would be critical for the survival of the host, as modulating the mDC response would influence the initiation of T-cell responses.

## Methods

### Ethics statement

Human peripheral blood was obtained from healthy donors with informed consent and was provided by the Etablissement Français du Sang (Lyon, France, agreement PLER/1‐1820‐05/05/14). Written informed consent was provided by all study participants.

### Virus and cells

VeroE6 cells were grown in DMEM supplemented with 0.5% penicillin-streptomycin and 5% fetal bovine serum (FBS, all from Invitrogen). A549 cells were grown in DMEM supplemented with 0.5% penicillin-streptomycin, 5% FBS and 1% Hepes (all from Invitrogen). Mopeia (AN21366 strain [16]) and Lassa (AV strain [47]) viruses were grown in VeroE6 cells at 37°C, with 5% CO_2_. Viral supernatants were harvested and used as the virus stock and the absence of mycoplasma was confirmed. LASV and MOPV titers were determined by plaque immunoassays as described below. All experiments with LASV were carried out in biosafety level 4 facilities (Laboratoire P4 Jean Merieux-Inserm, Lyon).

The reverse genetics systems of LASV and MOPV rely on a four-plasmid strategy, as described in [24], with the rescue procedure of recombinant viruses. Lassa virus with a non-functional exonuclease domain (LASV_NP ExoN_) was also obtained by reverse genetics and its characteristics are described in [24,25]. We obtained LASV and MOPV chimeras by first generating plasmids coding for the S or L segment and depleted for an ORF (or intergenic region of the S segment, IGRS). Briefly, plasmids encoding the S or L segment were amplified with primers, (i) allowing the complete amplification of the plasmid, except the target ORF and (ii) flanked with BsmBI restriction sites downstream of the start and upstream of the stop codons of the deleted ORF. PCR products were ligated and depleted segments used for the cloning of the heterologous ORF (or IGRS).

LASV-mCh and MOPV-mCh were modified to express the mCherry and LASV/MOPV NP proteins from a single gene. We used plasmids coding for the S segment of LASV or MOPV with a depleted NP (as described above). An insert containing the mCherry and NP ORFs, separated by a P2A self-cleavage site, was generated by overlapping PCR. This insert was cloned into the LASVΔNP or MOPVΔNP plasmid. MOPV and LASV with a FLAG-tagged Z protein were also obtained by reverse genetics. Sequences of all plasmid constructs were verified by sequencing.

### IFN-I neutralization

Autocrine and paracrine effects of the IFN-I response were neutralized (Fig 2) by treating mDCs with cocktails of neutralizing antibodies. The anti-IFN cocktail contained antibodies neutralizing IFNAR (MMHAR-2, 5 μg/mL), IFNα (MMHA-2, 2.5 μg/mL), and IFNβ (MMHB-3, 2.5 μg/mL), all from PBL Assay Science. In the control condition, the cocktail contained corresponding control isotype antibodies, IgG1 (5 μg/mL) and IgG2a (5 μg/mL), both from ThermoFisher Scientific. One dose of antibody cocktail was administrated to the cells at the time of infection. Half a dose was added to the cell culture 1 and 3 dpi. Cells were infected at a MOI = 0.1 by wild type or mCherry-expressing MOPV or LASV. For MOPV_WT_- and LASV_WT_-infected mDCs, small volumes of supernatant were harvested at various times post-infection and titrated. For mCherry-expressing MOPV and LASV, mCherry fluorescence was measured by fluorescence microscopy with Leica DMIRB.

### Virus titration

VeroE6 cells were infected with sequential dilutions of supernatant and maintained for six days with Carboxy-methyl-cellulose (1.6%) (BDH Laboratory Supplies) in DMEM supplemented with 2% FBS. Infectious foci were detected by incubation with monoclonal antibodies directed against MOPV and LASV (mAbs L52-54-6A, L53-237-5 and YQB06-AE05, generously provided by Dr P. Jahrling, USAM- RIID, Fort Detrick, MD), followed by PA-conjugated goat polyclonal anti-mouse IgG (Sigma-Aldrich).

### Cell purification

PBMCs were isolated by Ficoll (GE Healthcare) centrifugation from the blood of consenting healthy donors provided by the Etablissement Français du Sang (Lyon, France). mDCs were isolated by negative selection using the Myeloid Dendritic Cell Isolation kit (Miltenyi Biotech). mDCs were maintained in RPMI 1640 Glutamax I, 0.5% penicillin- streptomycin, 10 mM HEPES, 1% nonessential amino acids (full RPMI), and 10% FBS (all from Invitrogen). mDCs were infected at a MOI = 2 or treated with pIC (Invitrogen) at 150 μg/mL for a positive control of activation.

For mDC-T cell coculture, autologous plasma (AP) was heated for 30 min at 56°C and centrifuged for 20 min at 1,200 x g before use. mDCs were isolated from 75 to 80% of the PBMCs (as described above) and T cells from 20 to 25%. For T cells, peripheral blood lymphocytes (PBLs) were isolated by centrifugation on 50% Percoll (GE Healthcare) in phosphate-buffered saline (PBS). PBLs were washed three times in full RPMI supplemented with 4% AP. B cells were depleted using CD19 antibodies coupled to immunomagnetic beads (Dynal). T cells and mDCs were maintained overnight in full RPMI supplemented with 1 mM sodium pyruvate (Invitrogen) and 10% AP. mDCs were harvested and infected at a MOI = 1 for 1 h. Supernatants of uninfected vero cells were used for the mock condition. Infected mDCs were added to T cells at a ratio of one mDC to 10 T-cells.

For intracellular viral staining, purified mDCs, mDC-T coculture and A549 cells were infected with recombinant MOPV and LASV expressing Z-FLAG. mDCs were infected at MOI = 1 and cultures alone or with T cells at a ratio of one mDC to 10 T-cells. A549 cells were infected at MOI = 0.1.

Replicates of all experiments were performed with blood from different donors.

### RT-qPCR

To quantify cellular mRNA, cells were harvested and centrifuged. RNA from the cell pellets were extracted using the RNeasy kit and DNAse I digestion (both from Qiagen), followed by a second DNAse digestion (Ambion). cDNAs were randomly reverse transcribed using SuperScript III reverse transcriptase, Oligo (dT) 12–18 primers, and RNAse OUT ribonuclease inhibitors (all from Invitrogen). Cellular genes expression was assessed using the Taqman Universal master mix and Taqman commercial primers and probes for FasL, IFNα6, IFNα8, IFNγ, IL-15, IL-18, TNFα, TNFβ, TRAIL, and CXCL10 (Life Technologies). For the IFN-I genes, we used the following primers and probes: 5’-GTGGTGCTCAGCTGCAAGTC-3’ (sense), 5’-TGTGGGTCTCAGGGAGATCAC-3’ (antisense) and 5’-AGCTGCTCTCTGGGC-3’ (probe) for IFNα1; 5’-CAGTCTAGCAGCATCTGCAACAT-3’ (sense), 5’-GGAGGGCCACCAGTAAAGC-3’ (antisense) and 5’-ACAATGGCCTTGACCTT-3’ (probe) for IFNα2; 5’-TCTCCACGACAGCTCTTTCCA-3’ (sense), 5’-ACACTGACAATTGCTGCTTCTTTG-3’ (antisense) and 5’-AACTTGCTTGGATTCCT-3’ (probe) for IFNβ. GAPDH mRNAs were amplified using commercial primers and probes (Applied Biosystems) to normalize the results. Relative mRNA levels were calculated as 2^−ΔCt^, with Ct the cycle threshold and ΔCt = [gene Ct]–[GAPDH Ct].

For viral genome quantification, viral RNAs were extracted from culture supernatants using the QIAamp Viral RNA Mini Kit (Qiagen). Viral genomes were quantified using the EurobioGreen qPCR Mix Lo-ROX (Eurobio). Primers targeted MOPV NP (5’-CTTTCCCCTGGCGTGTCA-3’ and 5’-GAATTTTGAAGGCTGCCTTGA-3’) or LASV NP (5’-CTCTCACCCGGAGTATCT-3’ and 5’-CCTCAATCAATGGATGGC-3’). We transcribed the pGEM plasmid, coding for fragments of the MOPV and LASV NP gene (including the sequence amplified by PCR), *in vitro*, using the Riboprobe *in vitro* Transcription System (Promega), to obtain RNA standards. Viral genomes were quantified (by copy number) by comparing our samples with sequential dilutions of these standards. All runs were performed in duplicate using a LightCycler480 (Roche).

### Transcriptomics

mDCs were infected at a MOI = 1 by LASV or MOPV or were uninfected and incubated for 24 h at 37°C, 5% CO2. Cells were centrifuged and RNA from the cell pellets extracted using the RNeasy kit and DNAse I digestion (both from Qiagen), followed by a second DNAse digestion (Ambion). Experiments were replicated three times with cells from different donors. RNA quantity and quality were assed using the Agilent RNA 6000 nano kit and 2100 Bioanalyzer (Agilent Technologies).

Transcriptome sequencing and bioinformatic analyses were performed by GATC (Constance, Germany). cDNAs were synthesized by random priming of poly-A RNAs. Pair-end, 2x50 bp read-length illumina sequencing was performed on cDNA libraries, with a minimum of 30 million read pairs per sample. Pathway analysis was performed with Ingenuity Pathway Analysis software (Qiagen) and heatmaps were made using R.

### Flow cytometry

For mDC activation, mDCs were harvested 24 hpi, washed, and the pellets suspended in PBS complemented with 5% pooled human plasma. We incubated cells for 30 min at 4°C with Lin1-FITC, CD83(HB15e)-PE, CD40(5C3)-APC-H7 (BD Biosciences), CD11c(BU15)-PeCy5, CD86(HA5.2B7)-PeCy7, CD80(MAB104)-APC-AlexaFluor750, HLADR(Immu-357)-KromeOrange (Beckman Coulter), and/or CD253(RIK2.1)-APC (Miltenyi Biotech).

For mDC-T coculture, mDC activation was assessed at 48 hpi (using the same protocol as for mDCs alone). T-cell activation was assessed 2, 12, and 15 dpi. Cells were harvested, washed, and the pellets suspended in PBS complemented with 5% pooled human plasma. We incubated cells for 30 min a 4°C with CD4(SFCI12T4D11)-PECy7, CD3(UCHT1)-KromeOrange (Beckman Coulter), CD69(FN50)-PeCy7, CD4(RPA-T4)- AlexaFluor647, and/or CD8(RPA-T8)-BV421 (BD Biosciences). The expression of intracellular proteins was analyzed by treating the cells with the FoxP3 Staining Buffer Set and FcR Blocking Reagent, human (Miltenyi Biotech), according to the manufacturer’s instructions, and incubating them with Perforin(δG9)-FITC, GrzB(GB11)-PE, and/or Ki67(B56)-PerCPCy5.5 (BD Biosciences).

For staining of intracellular virus, purified mDCs and mDC-T cocultures were harvested at 2, 5, and 8 dpi and centrifuged. The cell pellets were suspended in PBS complemented with 5% pooled human plasma and incubated for 30 min a 4°C with Lin1-FITC, CD8(RPA-T8)-BV421 (BD Biosciences), CD11c(BU15)-PeCy5, CD4(SFCI12T4D11)-PECy7, CD3(UCHT1)-KromeOrange and/or HLADR(Immu-357)-KromeOrange (Beckman Coulter). Cells were treated with the FoxP3 Staining Buffer Set and FcR Blocking Reagent, human (Miltenyi Biotech), according to the manufacturer’s instructions, and incubated with anti-DYKDDDDK-APC antibody (Miltenyi Biotech).

The fluorescence of paraformaldehyde-fixed cells was measured using a Gallios flow cytometer (Beckman Coulter). Data were analyzed using Kaluza software (Beckman Coulter, version 1.2). DCs were gated as Lin1^-^/HLA-DR^+^ cells, and CD11c was used to confirm their phenotype. After phenotypic selection, based on FSC/SSC, CD4 and CD8 T cells were gated as CD3^+^/CD4^+^ and CD3^+^/CD8^+^ cells, respectively. See S7 Fig for more details.

### Luminex

mDCs were harvested 24 hpi and the culture medium collected. Fifty cytokines were quantified in the samples using the Milliplex map kit Human Cytokine/Chemokine Magnetic Bead Panel (PX38) and Human Cytokine/Chemokine Magnetic Bead Panel IV (Merck Millipore). Runs were performed with a Magpix Luminex (Merck Millipore).

### Western blot

VeroE6 cells infected with MOPV and LASV chimeras (MOI = 0.01) were lysed in Laemmli buffer (Bio-Rad) at 4 dpi. Heat-denaturated proteins were loaded and separated on 4–15% gradient precast gels and transferred onto PVDF membranes before staining. Samples were immunoblotted with primary antibodies against LASV GP1, LASV NP or LASV/MOPV Z, anti-mouse or anti-rabbit antibody conjugated to peroxydase (Jackson ImmunoResearch) and SuperSignal West Dura Extended Duration Substrate (ThermoFisher Scientific). Actin was used as a control for protein extraction and staining.

### Statistical analysis

The mean and standard error of the mean (SEM) for each set of data were calculated using R. Graphs were generated using SigmaPlot (SyStat Software Inc). Microscopy images were analyzed with AxioVision software (Zeiss, version 4.9).

Results between the various infection conditions (mock, pIC, MOPV, LASV …) were compared by performing Wilcoxon tests with R. Replication kinetics (in Fig 2A) were compared performing Repeated-Measures ANOVA followed by All Pairwise Multiple Comparison Procedures (Holm-Sidak method) using SigmaPlot. Differences between two sets of data were considered to be significant for p < 0.05.

## Supporting information

S1 Fig mDC response to MOPV and LASV. (A) mDCs were infected with MOPV (MOI = 2) or were uninfected and cellular RNA collected every 8 hpi. Quantification of IFN-I and TNFα mRNA was normalized to GAPDH expression. Data plotted are the fold change in MOPV-infected mDCs relative to uninfected mDCs. (B-C) mDCs were cultured for 24 h with MOPV or LASV (MOI = 2). Viral genomes in culture medium (B) or cell pellet (C) were quantified by RT-qPCR. (D) mDCs were cultured for 24 h with culture medium (mock), MOPV, or LASV (MOI = 1). Cellular mRNAs from three independent experiments were quantified using poly-A amplification and next-generation sequencing. Data shown are the differential expression of genes from the "dendritic cell maturation" pathway (from Ingenuity Pathway Analysis). Genes shown in this figure had significant differences of expression (adjusted p < 0.05).
(TIF)

S2 Fig MOPV and LASV infection of mDCs in coculture with T cells. (A-B) mDCs were infected with MOPV or LASV (MOI = 1) and cultured with T cells. Culture medium (A) was collected at day 2, 5, 8 and 12 post-infection, and cells (B) were collected at day 1, 2, 5, 8, 12 and 15 post-infection. Viral genomes in culture medium (A) or cell pellets (B) were quantified by RT-qPCR. (C) mDCs were infected with Z-tagged MOPV or LASV (MOI = 1) or uninfected (mock), and cultured with or without T cells (mDC alone and mDC in coculture, respectively). 2, 5 or 8 dpi, mDCs positive for the Z protein were quantified by flow cytometry. A549 cells infected with Z-tagged MOPV or LASV (MOI = 0.1) for 1 or 2 days were used as a control.
(TIF)

S3 Fig MOPV and LASV infection of T cells. For the “LT in coculture” condition, mDCs were infected with Z-tagged MOPV or LASV (MOI = 1) or uninfected (mock), and cultured with T cells. For the “LT” condition, purified T cells were infected with Z-tagged MOPV or LASV (MOI = 0.1) or uninfected (mock). 1, 2, 5 or 8 dpi, CD4 (A) and CD8 (B) T cells positive for the Z protein were quantified by flow cytometry. A549 cells infected with Z-tagged MOPV or LASV (MOI = 0.1) for 1 or 2 days were used as a control.
(TIF)

S4 Fig Evolution of mDC-T cell coculture over time. (A) mDCs were infected with MOPV or LASV (MOI = 1) or were uninfected and cultured for 48 h with T cells. Quantification of IFN-I and CXCL10 mRNA is expressed as the gene/GAPDH ratio. (B-C) CD4 T cells were gated as CD3^+^/CD4^+^ cells (B) and CD8 T cells as CD3^+^/CD8^+^ cells (C). Cells positive for activation molecules were counted. Results are expressed as the percentage of positive CD4 (B) or CD8 (C) T cells. Data shown are the means and SEM of seven independent experiments. Statistical significance was assessed by the non-parametric Wilcoxon test and differences were considered to be significant for p < 0.05 (*), p < 0.01 (**), or p < 0.001 (***).
(TIF)

S5 Fig Verification of ORF exchanges between MOPV and LASV. VeroE6 cells were infected with wild type and chimeric viruses (MOI = 0.01) for 4 days. Culture medium was collected and the natures of the viral stocks were determined by next generation sequencing. Data show the coverage of the obtained sequences, using MOPV (A) or LASV (B) genome as a reference.
(TIF)

S6 Fig Characterization of MOPV and LASV chimeras. (A-B-C) VeroE6 cells were infected with wild type and chimeric viruses (MOI = 0.01) for 4 days. (A) Cells were lysed 4 dpi, and viral proteins were detected by western blot. The anti-GP antibody only recognizes LASV GP1. The anti-NP antibody better recognizes LASV NP compared to MOPV NP. The anti-Z antibody recognizes both LASV and MOPV Z. (B) Culture medium was collected from 0 to 4 dpi and viral titers were determined. Data shown represent the mean ± SEM of 3 independent experiments. (C) Viral genomes in the culture medium were quantified by RT-qPCR 4 dpi. Data shown represent the mean ± SEM of the viral genomes/viral titer ratio for 4 independent experiments. Black and grey bars correspond to viruses with the MOPV and LASV backbones, respectively. (D) mDCs were infected with wild type and chimeric viruses (MOI = 1) and cultured with T cells. Culture medium was collected at day 2, 5, 8 and 12 post-infection, and viral titers were determined. Data shown represent the mean ± SEM of 3 independent experiments.
(TIF)

S7 Fig Gating of mDCs and T cells by flow cytometry. (A) Gates used to identify purified mDCs (Fig 1B). Data shown here are for uninfected mDCs at 24 hpi. Part of the debris was eliminated on FSC-SSC (cells). SSCint/SSCtof was used to exclude doublets (single). Among the “single” gated cells, mDCs were gated as Lin1^-^/HLADR^+^ cells. Lin1^-^/HLADR^-^ contained mostly debris. Contaminating cells (Lin1^+^) represented less than 25% of the cells in all experiments and were mainly monocytes (CD14^+^/CD16^+^), B cells (CD20^+^), and T cells (CD3^+^). (B) Gates used to identify mDCs in mDC-T coculture (Fig 4B). Data shown here are for uninfected cocultures at 48 hpi. Part of the debris was eliminated on FSC-SSC (cells). SSCint/SSCtof was used to exclude doublets (single). Among the “single” gated cells, mDCs were gated as Lin1^-^/HLADR^+^ cells. (C) Gates used to identify CD4 and CD8 T cells in mDC-T coculture (Fig 4B). Data shown here are for uninfected cocultures at 48 hpi. Lymphocytes (Lympho) were selected based on phenotype using FSC-SSC. SSCint/SSCtof was used to exclude doublets (single). Among the “single” gated cells, CD4 T cells were gated as CD3^+^/CD4^+^ cells (lower right panel). CD8 T cells were gated as CD3^+^/CD8^+^ cells, and Natural Killer cells (NK) as CD3^-^/CD8^+^ cells (upper right panel).
(TIF)
