# Supplementary figures and images for: Lassa virus activates myeloid dendritic cells but suppresses their ability to stimulate T cells
Source: PLoS Pathog. 2018 Nov 12;14(11):e1007430. doi: 10.1371/journal.ppat.1007430 (PMC6258464; doi:10.1371/journal.ppat.1007430)

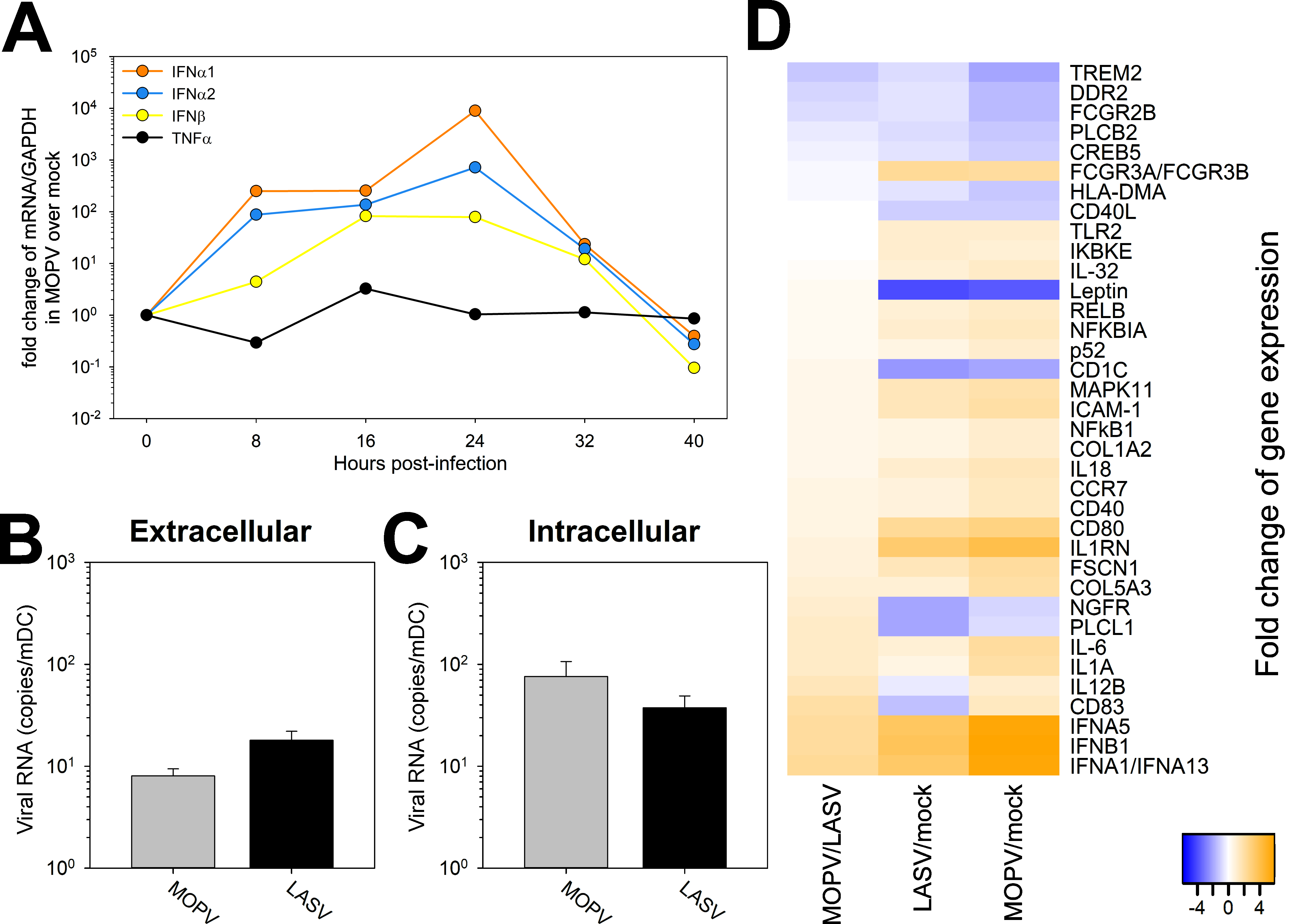

Supplement: S1 Fig — (A) mDCs were infected with MOPV (MOI = 2) or were uninfected and cellular RNA collected every 8 hpi. Quantification of IFN-I and TNFα mRNA was normalized to GAPDH expression. Data plotted are the fold change in MOPV-infected mDCs relative to uninfected mDCs. (B-C) mDCs were cultured for 24 h with MOPV or LASV (MOI = 2). Viral genomes in culture medium (B) or cell pellet (C) were quantified by RT-qPCR. (D) mDCs were cultured for 24 h with culture medium (mock), MOPV, or LASV (MOI = 1). Cellular mRNAs from three independent experiments were quantified using poly-A amplification and next-generation sequencing. Data shown are the differential expression of genes from the "dendritic cell maturation" pathway (from Ingenuity Pathway Analysis). Genes shown in this figure had significant differences of expression (adjusted p < 0.05). (TIF) [file ppat.1007430.s001.tif]

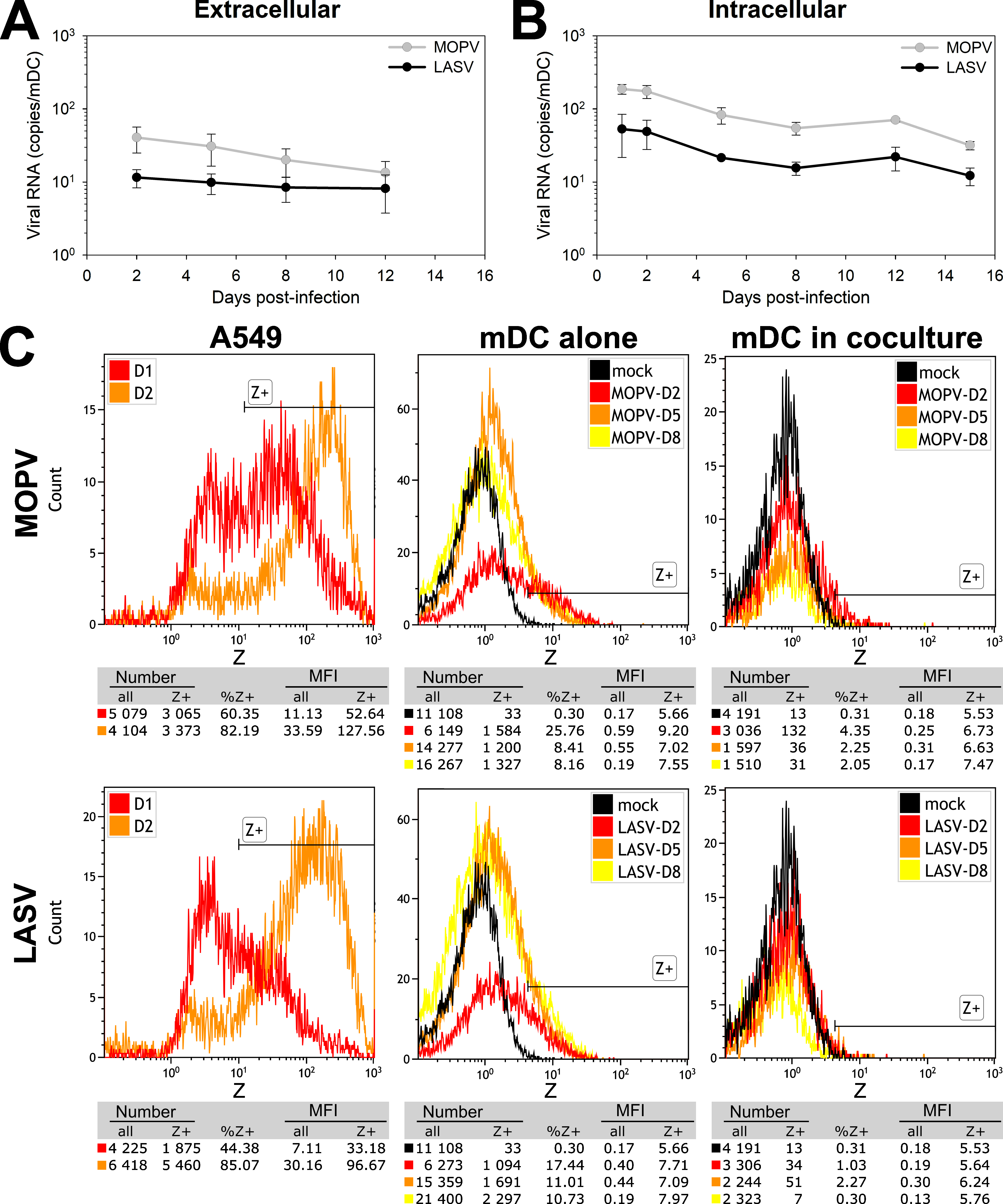

Supplement: S2 Fig — (A-B) mDCs were infected with MOPV or LASV (MOI = 1) and cultured with T cells. Culture medium (A) was collected at day 2, 5, 8 and 12 post-infection, and cells (B) were collected at day 1, 2, 5, 8, 12 and 15 post-infection. Viral genomes in culture medium (A) or cell pellets (B) were quantified by RT-qPCR. (C) mDCs were infected with Z-tagged MOPV or LASV (MOI = 1) or uninfected (mock), and cultured with or without T cells (mDC alone and mDC in coculture, respectively). 2, 5 or 8 dpi, mDCs positive for the Z protein were quantified by flow cytometry. A549 cells infected with Z-tagged MOPV or LASV (MOI = 0.1) for 1 or 2 days were used as a control. (TIF) [file ppat.1007430.s002.tif]

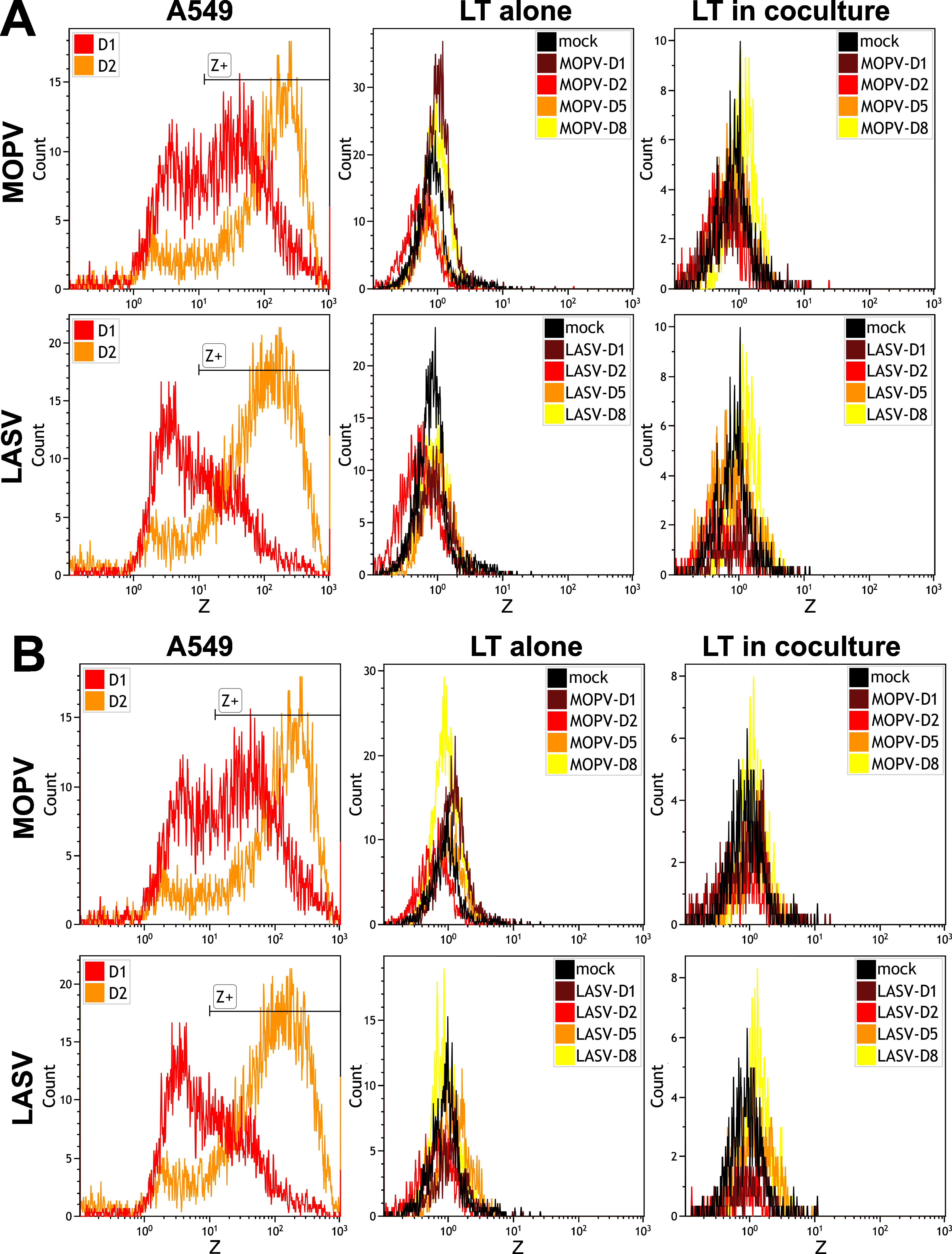

Supplement: S3 Fig — For the “LT in coculture” condition, mDCs were infected with Z-tagged MOPV or LASV (MOI = 1) or uninfected (mock), and cultured with T cells. For the “LT” condition, purified T cells were infected with Z-tagged MOPV or LASV (MOI = 0.1) or uninfected (mock). 1, 2, 5 or 8 dpi, CD4 (A) and CD8 (B) T cells positive for the Z protein were quantified by flow cytometry. A549 cells infected with Z-tagged MOPV or LASV (MOI = 0.1) for 1 or 2 days were used as a control. (TIF) [file ppat.1007430.s003.tif]

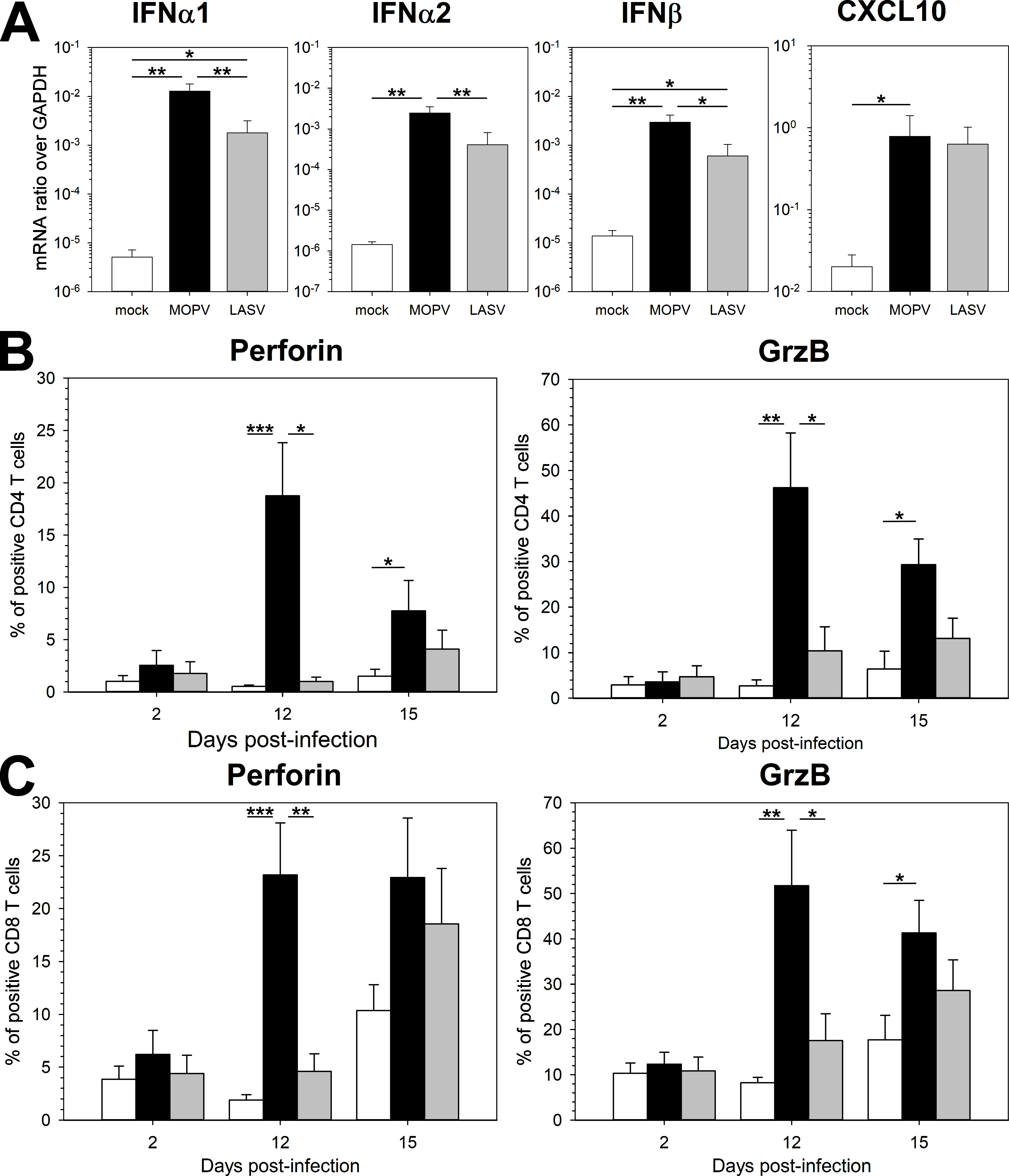

Supplement: S4 Fig — (A) mDCs were infected with MOPV or LASV (MOI = 1) or were uninfected and cultured for 48 h with T cells. Quantification of IFN-I and CXCL10 mRNA is expressed as the gene/GAPDH ratio. (B-C) CD4 T cells were gated as CD3+/CD4+ cells (B) and CD8 T cells as CD3+/CD8+ cells (C). Cells positive for activation molecules were counted. Results are expressed as the percentage of positive CD4 (B) or CD8 (C) T cells. Data shown are the means and SEM of seven independent experiments. Statistical significance was assessed by the non-parametric Wilcoxon test and differences were considered to be significant for p < 0.05 (*), p < 0.01 (**), or p < 0.001 (***). (TIF) [file ppat.1007430.s004.tif]

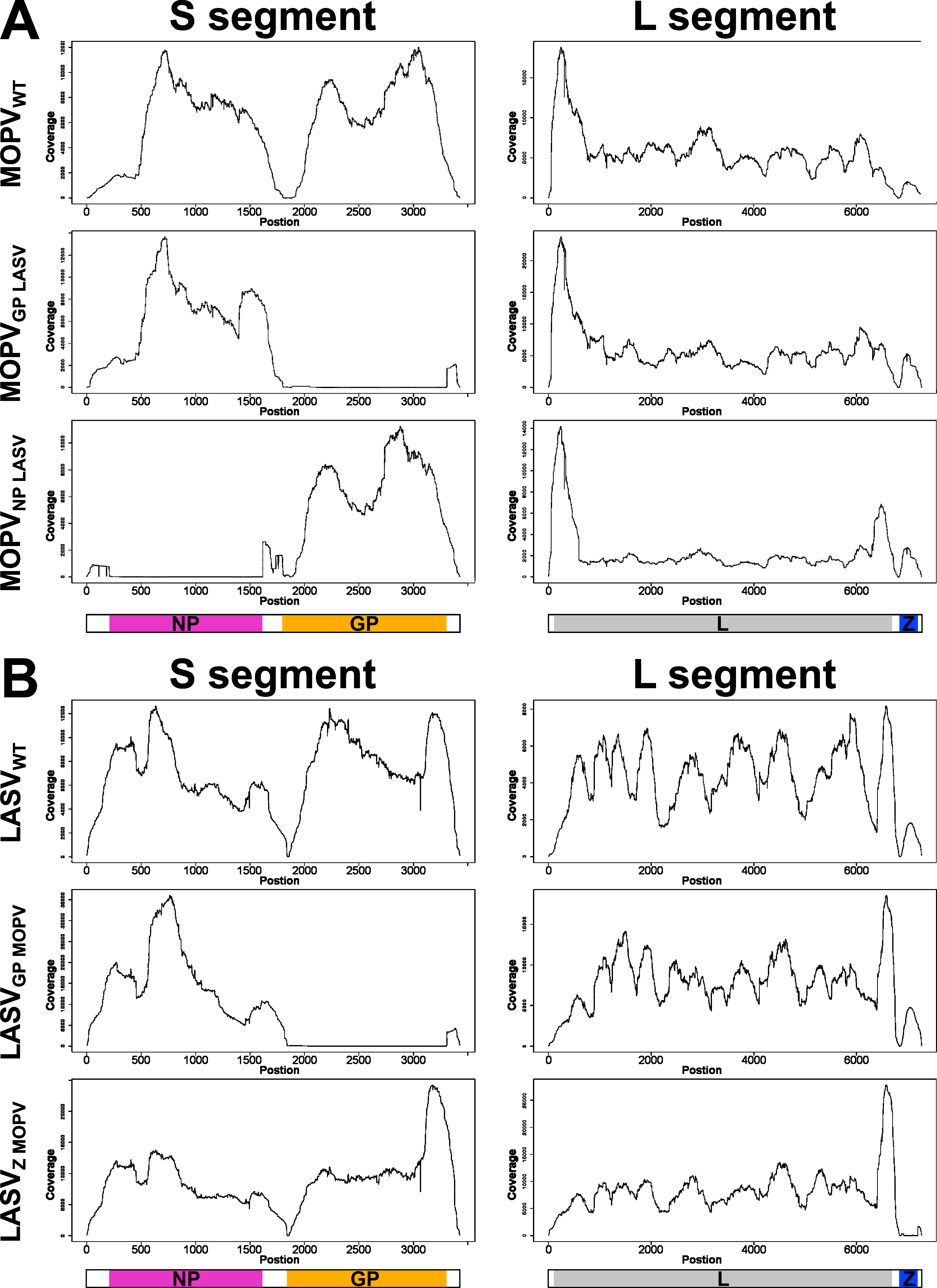

Supplement: S5 Fig — VeroE6 cells were infected with wild type and chimeric viruses (MOI = 0.01) for 4 days. Culture medium was collected and the natures of the viral stocks were determined by next generation sequencing. Data show the coverage of the obtained sequences, using MOPV (A) or LASV (B) genome as a reference. (TIF) [file ppat.1007430.s005.tif]

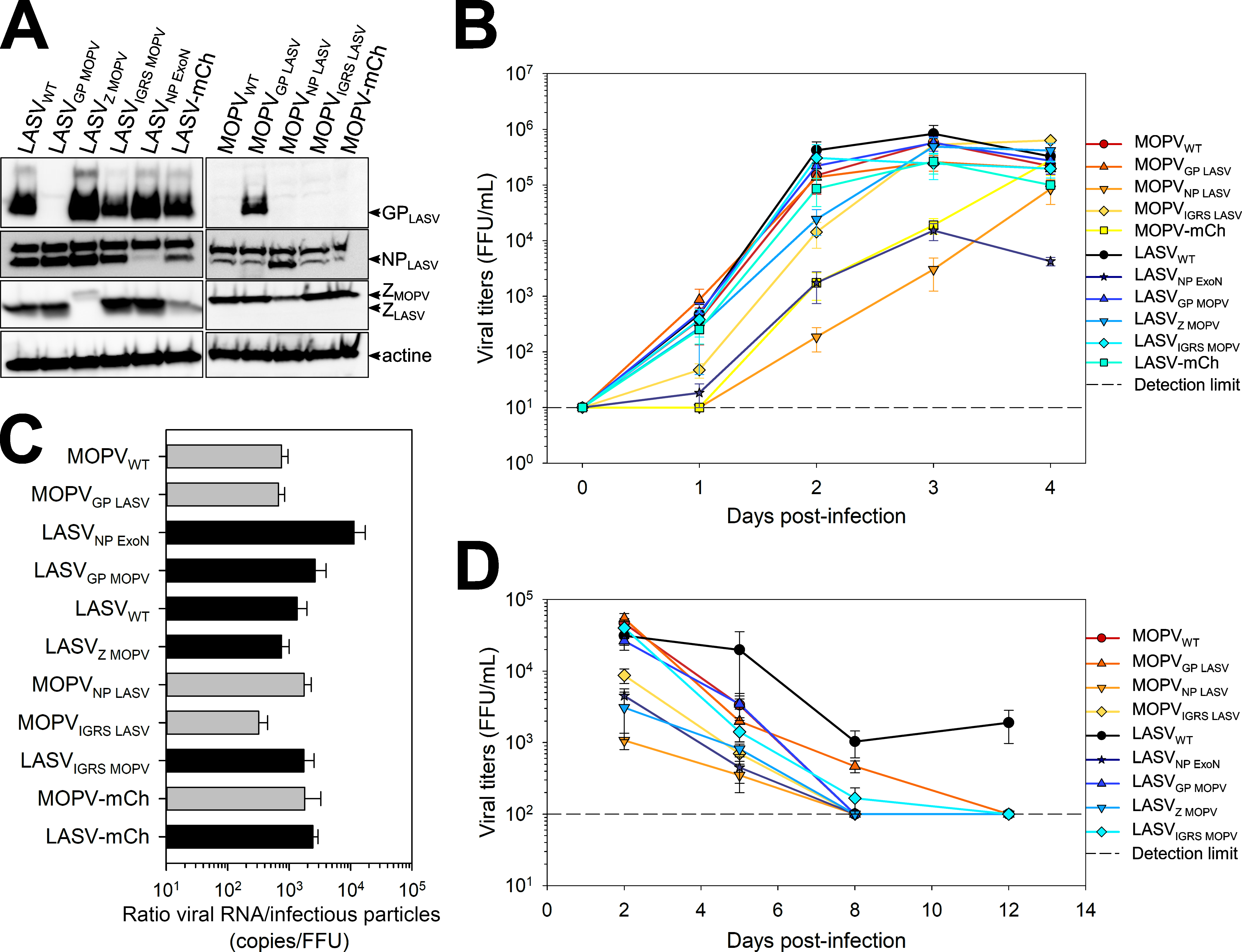

Supplement: S6 Fig — (A-B-C) VeroE6 cells were infected with wild type and chimeric viruses (MOI = 0.01) for 4 days. (A) Cells were lysed 4 dpi, and viral proteins were detected by western blot. The anti-GP antibody only recognizes LASV GP1. The anti-NP antibody better recognizes LASV NP compared to MOPV NP. The anti-Z antibody recognizes both LASV and MOPV Z. (B) Culture medium was collected from 0 to 4 dpi and viral titers were determined. Data shown represent the mean ± SEM of 3 independent experiments. (C) Viral genomes in the culture medium were quantified by RT-qPCR 4 dpi. Data shown represent the mean ± SEM of the viral genomes/viral titer ratio for 4 independent experiments. Black and grey bars correspond to viruses with the MOPV and LASV backbones, respectively. (D) mDCs were infected with wild type and chimeric viruses (MOI = 1) and cultured with T cells. Culture medium was collected at day 2, 5, 8 and 12 post-infection, and viral titers were determined. Data shown represent the mean ± SEM of 3 independent experiments. (TIF) [file ppat.1007430.s006.tif]

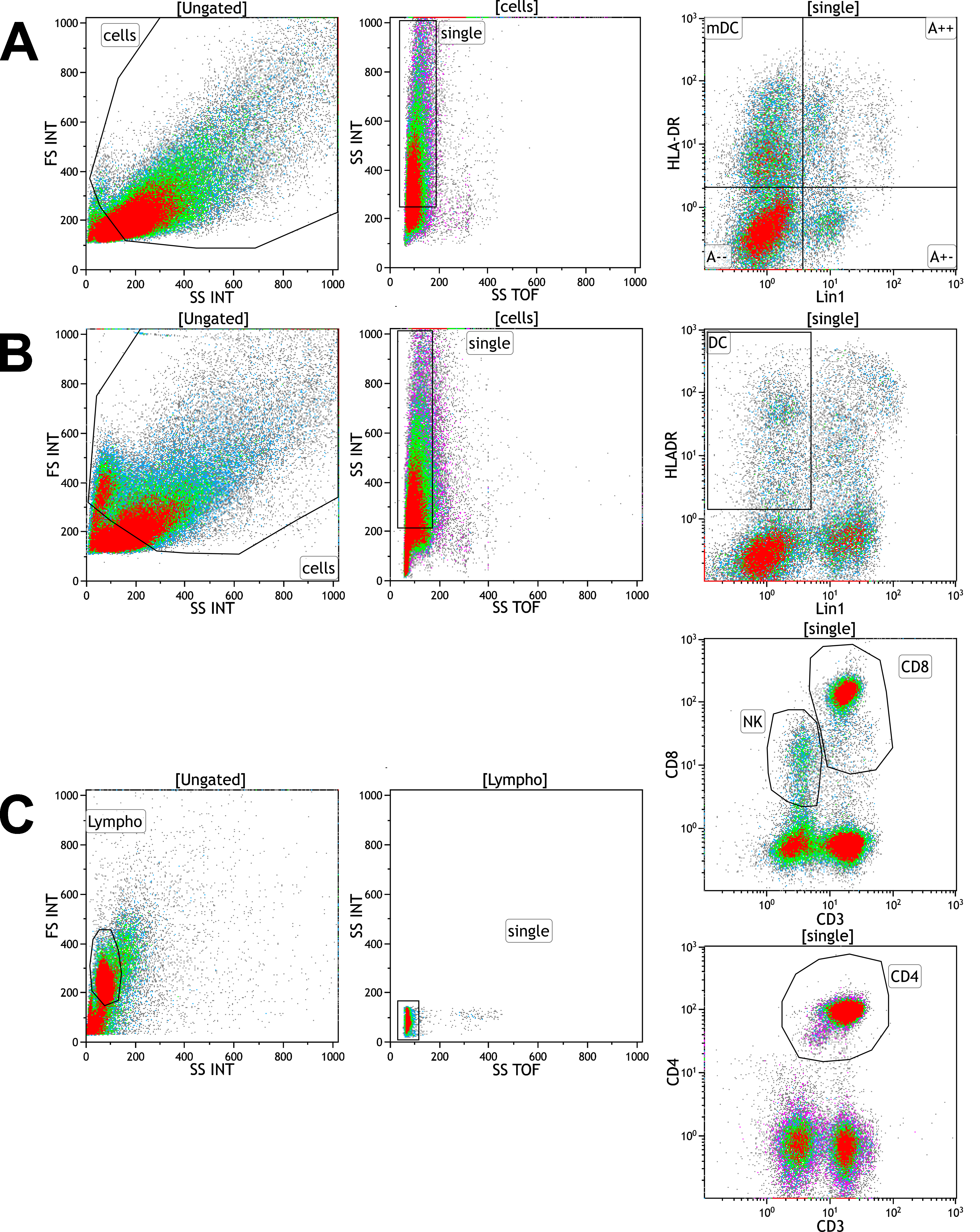

Supplement: S7 Fig — (A) Gates used to identify purified mDCs (Fig 1B). Data shown here are for uninfected mDCs at 24 hpi. Part of the debris was eliminated on FSC-SSC (cells). SSCint/SSCtof was used to exclude doublets (single). Among the “single” gated cells, mDCs were gated as Lin1-/HLADR+ cells. Lin1-/HLADR- contained mostly debris. Contaminating cells (Lin1+) represented less than 25% of the cells in all experiments and were mainly monocytes (CD14+/CD16+), B cells (CD20+), and T cells (CD3+). (B) Gates used to identify mDCs in mDC-T coculture (Fig 4B). Data shown here are for uninfected cocultures at 48 hpi. Part of the debris was eliminated on FSC-SSC (cells). SSCint/SSCtof was used to exclude doublets (single). Among the “single” gated cells, mDCs were gated as Lin1-/HLADR+ cells. (C) Gates used to identify CD4 and CD8 T cells in mDC-T coculture (Fig 4B). Data shown here are for uninfected cocultures at 48 hpi. Lymphocytes (Lympho) were selected based on phenotype using FSC-SSC. SSCint/SSCtof was used to exclude doublets (single). Among the “single” gated cells, CD4 T cells were gated as CD3+/CD4+ cells (lower right panel). CD8 T cells were gated as CD3+/CD8+ cells, and Natural Killer cells (NK) as CD3-/CD8+ cells (upper right panel). (TIF) [file ppat.1007430.s007.tif]
